# A burden of fluid, sodium, and chloride due to intravenous fluid therapy in patients with respiratory support: a post-hoc analysis of a multicenter cohort study

**DOI:** 10.1186/s13613-022-01073-x

**Published:** 2022-10-22

**Authors:** Masaaki Sakuraya, Shodai Yoshihiro, Kazuto Onozuka, Akihiro Takaba, Hideto Yasuda, Nobuaki Shime, Yuki Kotani, Yuki Kishihara, Natsuki Kondo, Kosuke Sekine, Keita Morikane, the AMOR-VENUS study group, Hideto Yasuda, Ryohei Yamamoto, Yoshihiro Hayashi, Yuki Kotani, Yuki Kishihara, Natsuki Kondo, Kosuke Sekine, Nobuaki Shime, Keita Morikane, Takayuki Abe, Toru Takebayashi, Mikihiro Maeda, Takuya Shiga, Taku Furukawa, Mototaka Inaba, Sachito Fukuda, Kiyoyasu Kurahashi, Sarah Murakami, Yusuke Yasumoto, Tetsuro Kamo, Masaaki Sakuraya, Rintaro Yano, Toru Hifumi, Masahito Horiguchi, Izumi Nakayama, Masaki Nakane, Kohei Ota, Tomoaki Yatabe, Masataka Yoshida, Maki Murata, Kenichiro Fujii, Junki Ishii, Yui Tanimoto, Toru Takase, Tomoyuki Masuyama, Masamitsu Sanui, Takuya Kawaguchi, Junji Kumasawa, Norimichi Uenishi, Toshihide Tsujimoto, Kazuto Onozuka, Shodai Yoshihiro, Takakiyo Tatsumichi, Akihiko Inoue, Bun Aoyama, Moemi Okazaki, Takuya Fujimine, Jun Suzuki, Tadashi Kikuchi, Satomi Tone, Mariko Yonemori, Kenji Nagaoka, Naomi Kitano, Masaki Ano, Ichiro Nakachi, Ai Ishimoto, Misa Torii, Junichi Maehara, Yasuhiro Gushima, Noriko Iwamuro

**Affiliations:** 1 Department of Emergency and Intensive Care Medicine, JA Hiroshima General Hospital, Jigozen 1-3-3, Hiroshima, JA 738-8503 Japan; 2 Department of Pharmacy, Onomichi General Hospital, Hiroshima, Japan; 3 Pharmaceutical Department, JA Hiroshima General Hospital, Hiroshima, JA Japan; 4 Department of Emergency and Critical Care Medicine, Jichi Medical University Saitama Medical Center, Saitama, Japan; 5 Department of Clinical Research Education and Training Unit, Keio University Hospital Clinical and Translational Research Center (CTR), Tokyo, Japan; 6 Department of Emergency and Critical Care Medicine, Graduate School of Biomedical and Health Sciences, Hiroshima University, Hiroshima, Japan; 7 Department of Intensive Care Medicine, Kameda Medical Center, Chiba, Japan; 8 Department of Intensive Care Medicine, Chiba Emergency Medical Center, Chiba, Japan; 9 Department of Medical Engineer, Kameda Medical Center, Chiba, Japan; 10 Division of Clinical Laboratory and Infection Control, Yamagata University Hospital, Yamagata, Japan

**Keywords:** Fluid therapy, Fluid creep, Intravenous fluid, Hypoxemic respiratory failure

## Abstract

**Background:**

Fluid creep, including fluids administered as drug diluents and for the maintenance of catheter patency, is the major source of fluid intake in critically ill patients. Although hypoxemia may lead to fluid restriction, the epidemiology of fluid creep in patients with hypoxemia is unclear. This study aimed to address the burden due to fluid creep among patients with respiratory support according to oxygenation status.

**Methods:**

We conducted a post-hoc analysis of a prospective multicenter cohort study conducted in 23 intensive care units (ICUs) in Japan from January to March 2018. Consecutive adult patients who underwent invasive or noninvasive ventilation upon ICU admission and stayed in the ICU for more than 24 h were included. We excluded the following patients when no fluids were administered within 24 h of ICU admission and no records of the ratio of arterial oxygen partial pressure to fractional inspired oxygen. We investigated fluid therapy until 7 days after ICU admission according to oxygenation status. Fluid creep was defined as the fluids administered as drug diluents and for the maintenance of catheter patency when administered at ≤ 20 mL/h.

**Results:**

Among the 588 included patients, the median fluid creep within 24 h of ICU admission was 661 mL (25.2% of the total intravenous-fluid volume), and the proportion of fluid creep gradually increased throughout the ICU stay. Fluid creep tended to decrease throughout ICU days in patients without hypoxemia and in those with mild hypoxemia (*p* < 0.001 in both patients), but no significant trend was observed in those with severe hypoxemia (*p* = 0.159). Similar trends have been observed in the proportions of sodium and chloride caused by fluid creep.

**Conclusions:**

Fluid creep was the major source of fluid intake among patients with respiratory support, and the burden due to fluid creep was prolonged in those with severe hypoxemia. However, these findings may not be conclusive as this was an observational study. Interventional studies are, therefore, warranted to assess the feasibility of fluid creep restriction.

*Trial registration* UMIN-CTR, the Japanese clinical trial registry (registration number: UMIN 000028019, July 1, 2017).

**Supplementary Information:**

The online version contains supplementary material available at 10.1186/s13613-022-01073-x.

## Background

Intravenous fluid is commonly administered to critically ill patients. The aim of intravenous-fluid administration can be categorized into resuscitation and non-resuscitation (e.g., replacement and maintenance) [1]. Non-resuscitation fluids were reported to have a greater impact on cumulative fluid balance than resuscitation fluids [2, 3]. Observational studies have suggested that positive fluid balance is associated with poor outcomes in critically ill patients [4–6]. Furthermore, sodium intake may contribute to a prolonged mechanical ventilation duration [7], and chloride administration has also been reported to be a risk factor for the development of acute kidney injury [8]. These findings from observational studies have inspired research to investigate whether conservative fluid management can improve survival or reduce the incidence of organ dysfunction in critically ill patients [9–19].

The prevalence of fluids administered as drug diluents and for the maintenance of catheter patency, so-called fluid creep, has recently been investigated [2–4, 20, 21]. Fluid creep is also associated with the burden of fluid, sodium, and chloride. Replacement of normal saline with 5% dextrose for fluid creep potentially prevents unnecessary sodium and chloride administration [2, 20, 21].

Resuscitation is challenging in septic shock patients with hypoxemic respiratory failure, since hypoxemia is one of the conditions that discourage fluid resuscitation [22]. Furthermore, in patients with hypoxemic respiratory failure, fluid restriction has been a common strategy for a longer ventilator-free period [18, 19]. However, conservative fluid management in the previous studies involved the restriction of resuscitative, replacement, and maintenance fluids, but not fluid creep [9–19]. Fluid creep is a major source of fluid volume, sodium, and chloride in critically ill patients, who are administered many kinds of intravenous drugs [2, 3]. Severe hypoxemia is one of the triggers of deep sedation, which may increase the demand for vasoactive drugs [23–26]. Fluid creep may increase in such patients despite the attempts to decrease fluid intake. Therefore, it is warranted to reveal the epidemiology of fluid creep in patients with respiratory support according to oxygenation status.

In this study, we conducted a post-hoc analysis of the incidence and risk factors for phlebitis and complications due to peripheral venous catheters in critically ill patients (AMOR-VENUS study) [27], which was a prospective multicenter cohort study of the general intensive care unit (ICU) population. This analysis aimed to address the burden of volume, as well as the sodium and chloride burden due to fluid creep among patients with respiratory support.

## Methods

### Study design and setting

This study was a post-hoc analysis of the AMOR-VENUS study conducted in 23 ICUs in Japan from January 1 to March 31, 2018. This study was approved by the institutional review board or medical ethics committee of each institution (Approval number: 17–50). The requirement for informed consent was waived, and an opt-out recruitment method was employed. This study is reported in accordance with the Strengthening the Reporting of Observational Studies in Epidemiology statement [28].

### Study participants

Consecutive adult patients (aged ≥ 18 years) admitted to the ICU during the study period were included in the AMOR-VENOUS study. The exclusion criteria of the original data set were as follows: refusal to participate in the study, having no intravascular catheters during ICU stay, and physician’s discretion, that is, patients who were selected for exclusion by physicians (e.g., due to a short stay in the ICU). Of these patients, this post-hoc analysis included those who underwent invasive or noninvasive ventilation upon ICU admission. In addition, patients were excluded if they died or were discharged within 24 h of ICU admission, no fluids were administered within 24 h of ICU admission, or the ratio of arterial oxygen partial pressure to fractional inspired oxygen (P/F ratio) was not recorded.

### Variables and measurements

Baseline data included age, admission category (non-scheduled surgery, scheduled surgery, and medical emergency), the severity of illness (Acute Physiology and Chronic Health Evaluation II [APACHE-II] score [29] and Sequential Organ Failure Assessment [SOFA] score [30]), Charlson comorbidity index [31], presence of sepsis and acute kidney injury, P/F ratio, mortality, and lengths of ICU stay and hospitalization.

### Exposure and outcomes

The AMOR-VENUS data set includes all inserted intravenous catheters and intravenous fluids. Data on the type of fluids, drugs, drug vehicle, and rate of intravenous fluid administration were also collected. However, data on the aim of intravenous fluid, oral fluid intake, and output data were not collected. Using these data, we calculated the amounts of fluid, sodium, and chloride per 24 h. Fluids were classified as isotonic crystalloids (e.g., normal saline and Ringer’s solution), hypotonic crystalloids (e.g., 5% dextrose, glucose-containing fluids in place of electrolytes), nutrition fluids (which contained ≥ 10% dextrose or amino acids), colloids, blood products, or vehicles for drugs. The dosage of the liquid drug was defined as an unclassified fluid. Fluid creep was defined as fluids administered as drug diluents (antibiotics, sedatives and analgesics, vasoactive drugs, and any other drugs) and for the maintenance of catheter patency [2].

The following solutions were considered the fluids for maintenance of catheter patency, which were administered at ≤ 20 mL/h: isotonic crystalloids and hypotonic crystalloids [32]. Isotonic crystalloid was divided into three categories: resuscitation fluids when administered at a rate > 166 mL/h (equivalent to 1 L over 6 h) [2], fluids for catheter patency ≤ 20 mL/h, and maintenance fluids when administered in the range between both fluids. Hypotonic crystalloid was divided into two categories: fluids for catheter patency ≤ 20 mL/h and maintenance fluids > 20 mL/h. Fluid creep was divided based on drugs (antibiotics, sedatives and analgesics, and vasoactive drugs), and vehicles for other drugs and any fluids to maintain catheter patency were categorized as miscellaneous use. We evaluated the fluid therapy until 7 days from the ICU admission or discharge, whichever was shorter, according to the oxygenation status at ICU admission (patients without hypoxemia [P/F ratio > 300], patients with mild hypoxemia [150 < P/F ratio ≤ 300], and patients with severe hypoxemia [P/F ratio ≤ 150], which is considered a threshold for deep sedation with neuromuscular blockade and prone position) [23, 24]. All patients were followed-up until hospital discharge to assess hospital mortality.

### Statistical analysis

Data are expressed as medians with interquartile ranges (IQRs) or means with standard deviations for continuous variables and numbers with corresponding percentages for dichotomous variables. In all analyses, the number of cases with missing data was reported, and these cases were excluded from each analysis. Baseline characteristics were compared between the study groups. Continuous variables were compared using the unpaired *t* test, Mann–Whitney *U* test, one-way analysis of variance, or Kruskal–Wallis test, according to the data distribution. Dichotomous variables were analyzed using the chi-squared test or Fisher’s exact test. According to the patient’s oxygenation status, we evaluated the trend toward increasing or decreasing intravenous fluid, sodium, and chloride intake with longer ICU stays using the Jonckheere–Terpstra trend test. Multiple linear regression models were used to evaluate the relationship of septic shock and hypoxemic respiratory failure to the amount of intravenous fluid and fluid creep within 24 h of ICU admission, as well as sodium and chloride burden. Covariables known to be associated with fluid management in critically ill adults (i.e., admission category and underlying disease severity) were identified a priori and subsequently forced into the models [2, 33, 34]. In addition, we included cardiovascular disease and hypoxemic respiratory failure (P/F ratio ≤ 300) in the models to explore their relationship with fluid therapy. All statistical tests were two-sided, and statistical significance was set at *p* < 0.05. Statistical analyses were performed using Stata 15.1 (StataCorp LLC, College Station, TX, USA).

## Results

**Fig. 1:**
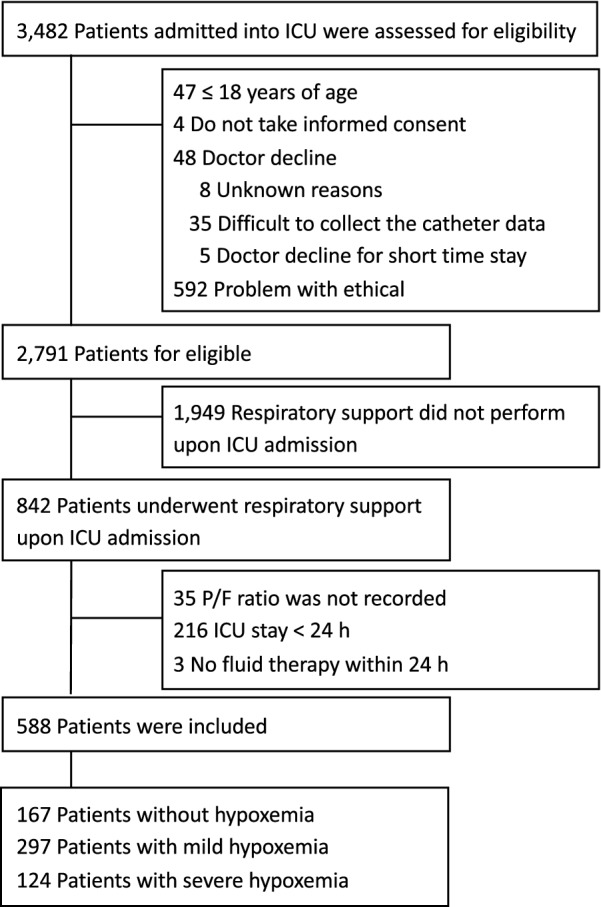
Patient flow diagram**.** Abbreviations: ICU, intensive care unit; P/F ratio, ratio of arterial oxygen partial pressure to fractional inspired oxygen

A total of 3482 patients were admitted to the ICU during the study period. Among these, 842 patients underwent mechanical or noninvasive ventilation upon ICU admission, and 588 were included in this analysis after applying the inclusion and exclusion criteria (Fig. 1). The patients were divided into the following three groups according to oxygenation: patients without hypoxemia (167 [28.4%]), patients with mild hypoxemia (297 [50.5%]), and patients with severe hypoxemia (124 [21.1%]). None of the patients was lost to follow-up until hospital discharge.

Patient demographics and clinical characteristics are presented in Tables 1 and 2, respectively. Of the 588 patients, 380 (64.6%) were men, and nearly half were medical patients (300 patients, 51.0%). Noninvasive ventilation was performed in 80 patients (13.6%), and 508 patients (86.4%) were intubated upon ICU admission. The median (IQR) P/F ratio was 226.5 (range, 162–317.5). ICU and hospital mortality rates were 7.5% and 17.4%, respectively.

**Table 1:** Characteristics of patient population, days of hospital stay, mortality

	All patients	Without hypoxemia (P/F ratio > 300)	Mild hypoxemia (150 < P/F ratio ≤ 300)	Severe hypoxemia (P/F ratio ≤ 150)	*P* value
	*N* = 588	*N* = 167	*N* = 297	*N* = 124	
Age, mean (SD), y	67.5 (15.7)	62.2 (19.4)	69.4 (13.6)	69.9 (12.8)	< 0.001
Male, *n* (%)	380 (64.6)	96 (57.5)	193 (65.0)	91 (73.4)	0.019
Body mass index, mean (SD), kg/m^2^	23.1 (4.3)	21.9 (3.5)	22.9 (21.1–25.8)	24.2 (4.4)	< 0.001
Patient admission category					< 0.001
Non-scheduled surgery, *n* (%)	138 (23.4)	43 (25.8)	73 (24.6)	22 (17.7)	
Scheduled surgery, *n* (%)	150 (25.5)	60 (35.9)	73 (24.6)	17 (13.7)	
Medical emergency, *n* (%)	300 (51.0)	64 (38.3)	151 (50.8)	85 (68.6)	
APACHE II score, median (IQR)	19 (13.5–24)	16 (11–22)	18 (14–24)	21 (17–27)	0.001
SOFA score, median (IQR)	7 (5–10)	5 (3–7)	8 (6–10)	8.5 (6.5–11)	0.001
Charlson comorbidity index, median (IQR)	4 (2–6)	3 (1–5)	5 (3–6)	4 (3–6)	< 0.001
Sepsis category					0.006
Sepsis, *n* (%)	48 (8.2)	8 (4.8)	26 (8.8)	14 (11.3)	
Septic shock, *n* (%)	74 (12.6)	11 (6.6)	43 (14.5)	20 (16.1)	
Acute kidney injury					< 0.001
Stage 1, *n* (%)	49 (8.3)	8 (4.8)	22 (7.4)	19 (15.3)	
Stage 2, *n* (%)	31 (5.3)	4 (2.4)	16 (5.4)	11 (8.9)	
Stage 3, *n* (%)	58 (9.9)	12 (7.2)	31 (10.4)	15 (12.1)	
Respiratory support^c^					0.004
Noninvasive ventilation, *n* (%)	80 (13.6)	12 (7.2)	43 (14.5)	25 (20.2)	
Invasive ventilation, *n* (%)	508 (86.4)	155 (92.8)	254 (85.5)	99 (79.8)	
P/F ratio^c^, median (IQR)	226.5 (162–317.5)	368 (335–418)	217 (187–256)	114.5 (90–132)	< 0.001
Length of ICU Stay^a^, median (IQR), h	108.1 (58.9–191.0)	68.7 (43.7–131.4)	110.3 (65.5–211.4)	141.8 (97.2–249.6)	0.001
ICU mortality, *n* (%)	44 (7.5)	9 (5.4)	18 (6.1)	17 (13.7)	0.019
Length of hospitalization^b^, median (IQR), d	31 (19–56)	26.5 (15–52)	32 (19–57)	35 (23–58)	0.032
Hospital mortality, *n* (%)	102 (17.4)	17 (10.2)	50 (16.8)	35 (28.2)	0.003

^a^Excluded 40 patients who were died during ICU stay

^b^Excluded 102 patients who were died during hospital stay

^c^At ICU admission

*APACHE* acute physiology and chronic health evaluation, *ICU* intensive care unit, *IQR* interquartile range, *P/F ratio* ratio of arterial oxygen partial pressure to fractional inspired oxygen, *SD* standard deviation, *SOFA* sequential organ failure assessment

**Table 2:** ICU admission category

	All patients	Without hypoxemia (P/F > 300)	Mild hypoxemia (150 < P/F ≤ 300)	Severe hypoxemia (P/F ≤ 150)
	*N* = 588	*N* = 167	*N* = 297	*N* = 124
Cardiology, *n* (%)	225 (38.3)	49 (29.3)	124 (41.8)	52 (41.9)
Pulmonary, *n* (%)	95 (16.2)	9 (5.4)	43 (14.5)	43 (34.7)
Gastrointestinal, *n* (%)	67 (11.4)	19 (11.4)	38 (12.8)	10 (8.1)
Neurology, *n* (%)	73 (12.4)	38 (22.8)	29 (9.8)	6 (4.8)
Sepsis, *n* (%)	34 (5.8)	6 (3.6)	21 (7.1)	7 (5.7)
Trauma, *n* (%)	20 (3.4)	10 (6.0)	8 (2.7)	2 (1.6)
Endocrine, *n* (%)	14 (2.4)	5 (3.0)	6 (2.0)	3 (2.4)
Haematology, *n* (%)	4 (0.7)	0 (0)	3 (1.0)	1 (0.8)
Urology, *n* (%)	5 (0.9)	1 (0.6)	4 (1.4)	0 (0)
Gynaecology, *n* (%)	3 (0.5)	2 (1.2)	1 (0.3)	0 (0)
Skin/soft tissue, *n* (%)	11 (1.9)	3 (1.8)	8 (2.7)	0 (0)
Others, *n* (%)	37 (6.3)	25 (15.0)	12 (4.0)	0 (0)

**Fig. 2:**
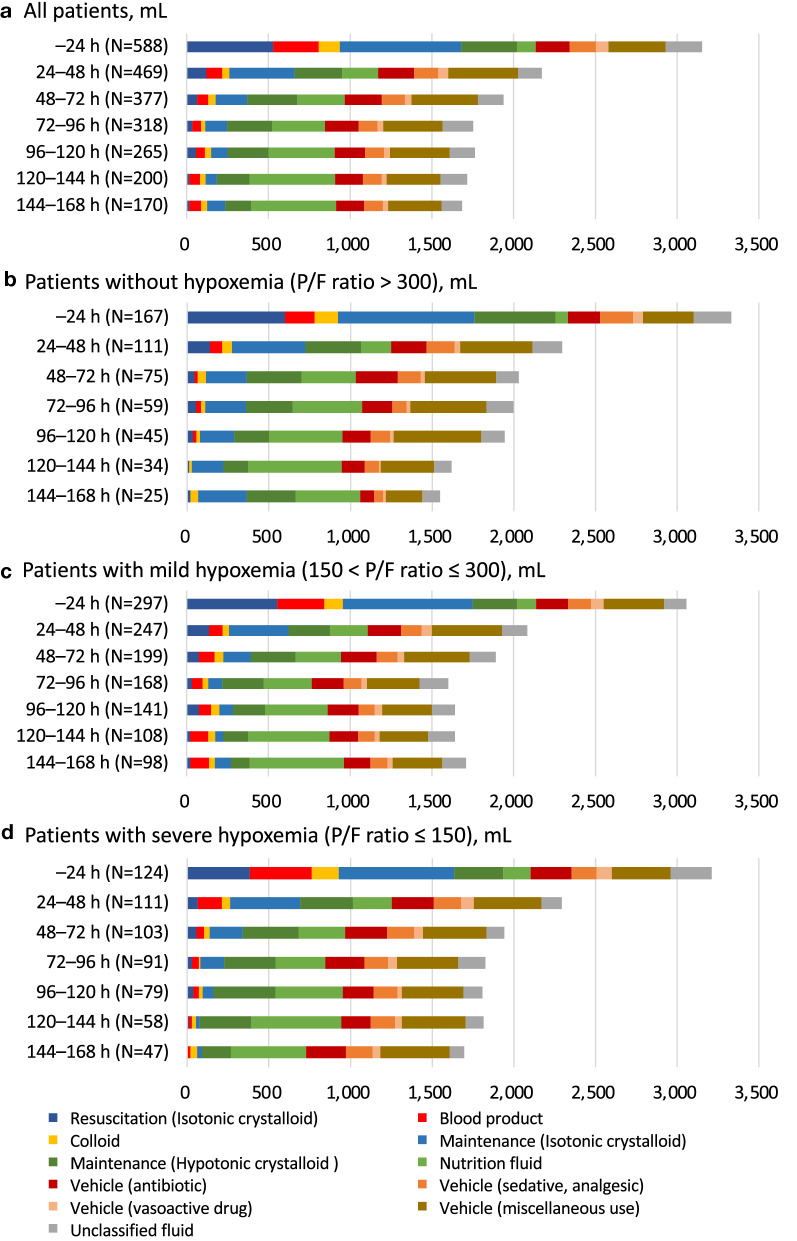
Daily intravenous fluids intake. **a** All patients; **b** patients without hypoxemia (P/F ratio > 300); **c** patients with mild hypoxemia (150 < P/F ratio ≤ 300); **d** patients with severe hypoxemia (P/F ratio ≤ 150). The dosage of the liquid drug was defined as an unclassified fluid. Abbreviations: P/F ratio, ratio of arterial oxygen partial pressure to fractional inspired oxygen

A mean total intravenous-fluid volume of 2662 (range, 1646–4059) mL was administered during the first 24 h after ICU admission, and the total amount of intravenous fluid gradually decreased but remained > 1000 mL per 24 h during the entire observation period (Fig. 2 and Additional file 1: Table S1). Isotonic crystalloid was the main intravenous fluid administered within 24 h of ICU admission, accounting for 40.3% of the cases. After 24 h, the amount of hypotonic fluid, especially for nutrition, and fluid creep increased and was the major source of intravenous fluid intake. In patients without hypoxemia, the crystalloid solution was still administered at approximately 20% of the total intravenous fluid, even in the latter part of the observation period; in contrast, it was lower in patients with severe hypoxemia. The daily sodium and chloride burdens of intravenous fluid are presented in Additional file 1: Tables S2 and S3, respectively. Both sodium and chloride were dosed at an estimated mean of 300 mEq within 24 h of ICU admission and subsequently decreased to approximately 100 mEq per 24 h (Additional file 1: Tables S2, S3).

**Fig. 3:**
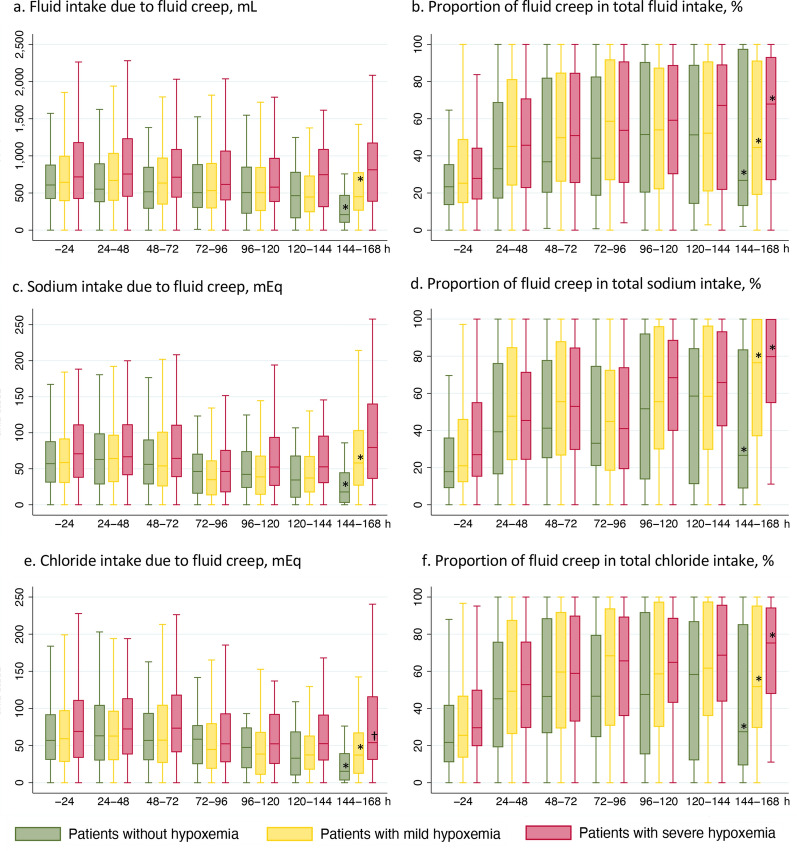
Daily intake of fluid, sodium, and chloride due to fluid creep. **a** Fluid intake due to fluid creep; **b** proportion of fluid creep in total fluid intake; **c** sodium intake due to fluid creep; **d** proportion of fluid creep in total sodium intake; **e** chloride intake due to fluid creep; **f** proportion of fluid creep in total chloride intake. * *P* for trend < 0.001; † *P* for trend < 0.01. There was no significant trend between intensive care unit days and the amount of fluid creep (*P* for trend = 0.159) and in sodium of fluid creep (*P* for trend = 0.105) in patients with severe hypoxemia

The median fluid creep during the first 24 h after ICU admission was 661 mL (range: 402–984 mL), constituting 25.2% of the total intravenous-fluid volume. After 24 h, fluid creep tended to decrease throughout ICU days in patients without hypoxemia and in those with mild hypoxemia (*P* for trend < 0.001 in both patients), but no significant trend was observed in those with severe hypoxemia (*P* for trend = 0.159) (Fig. 3). Meanwhile, significant trends were observed in the increase of the proportion of fluid creep in total intravenous-fluid volume among all patient groups (*P* for trend < 0.001). Similar trends were observed regarding the proportions of sodium and chloride due to fluid creep.

The results of the multiple linear regression model for the amount of intravenous fluid, sodium, and chloride burdens within 24 h of ICU admission after adjusting for the prespecified covariates are shown in Table 3 and Additional file 1: Table S4. Septic shock was associated with an increased fluid volume, sodium, and chloride burdens, except for sodium burden due to fluid creep. In contrast, hypoxemic respiratory failure was associated with a reduction in the total amount of intravenous fluids, sodium, and chloride, despite not being associated with the amount due to fluid creep.

**Table 3:** Estimates of the effects of septic shock and hypoxemic respiratory failure to a burden of fluid, sodium, and chloride within 24 h

	Septic shock (95% CI)	*P* value	Hypoxemic respiratory failure (95% CI)	*P* value
Fluid volume				
Total intravenous fluid, mL	1756.9 (1123.9 to 2390.0)	< 0.001	− 467.2 (− 913.8 to − 20.5)	0.040
Intravenous fluid due to fluid creep, mL	249.8 (47.6 to 452.0)	0.016	− 50.1 (− 192.8 to 92.5)	0.490
Sodium burden				
Total sodium burden, mEq	227.0 (143.1 to 310.9)	< 0.001	− 74.0 (− 133.2 to − 14.8)	0.014
Sodium burden due to fluid creep, mEq	14.0 (− 8.4 to 36.4)	0.220	− 0.9 (− 16.7 to 14.9)	0.913
Chloride burden				
Total chloride burden, mEq	194.8 (121.5 to 268.2)	< 0.001	− 61.4 (− 113.1 to − 9.7)	0.020
Chloride burden due to fluid creep, mEq	25.3 (2.5 to 48.2)	0.030	− 3.6 (− 19.7 to 12.6)	0.663

Adjusted by the following factors: sex, age, body mass index, emergency admission, cardiovascular disease, APACHE II score, SOFA score, Charlson comorbidity index. Multicollinearity using variance inflation factors was not detected among all covariates

*APACHE* acute physiology and chronic health evaluation, *CI* confidence interval, *SOFA* sequential organ failure assessment

**Fig. 4:**
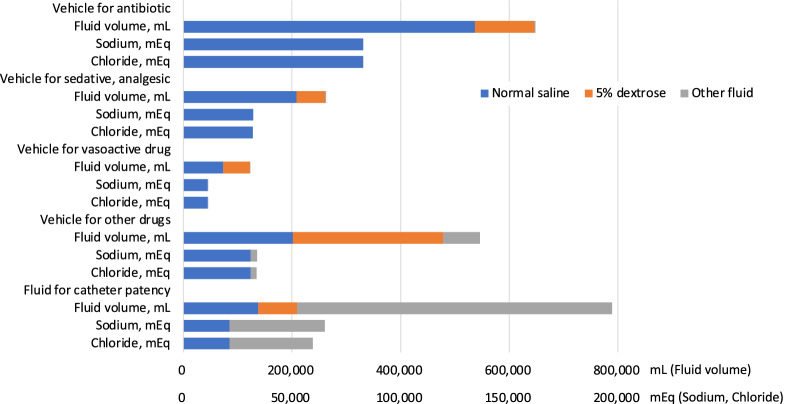
Details of total amount of fluid volume, sodium, and chloride burden due to fluid creep among the 588 patients. Each result was calculated from intravenous fluids administered in 588 patients for 2387 patient-days.

Among 588 patients with 2387 patient-days, the most used drug vehicle was normal saline (Fig. 4), with the vehicle for antibiotics accounting for the larger amount compared with other drug vehicles. The majority of fluids for catheter patency encompassed other crystalloids, followed by normal saline and 5% dextrose.

## Discussion

### Key findings

This post-hoc analysis of a prospective multicenter observational study investigated the epidemiology of intravenous fluid therapy among patients with respiratory support. Among the 588 included patients, fluid creep was a major source of fluid volume, sodium, and chloride burdens. Fluid creep tended to decrease throughout ICU days in patients without hypoxemia and in those with mild hypoxemia, but no significant trend was observed in those with severe hypoxemia. After adjusting for the prespecified confounding factors, septic shock was associated with most of the burdens; meanwhile, hypoxemic respiratory failure was associated with a reduction in the total amount of intravenous fluid, but not with fluid creep.

### Relationship with previous studies

Previous studies reported that the median daily amount of fluid creep was ≥ 600 mL, constituting approximately 35% of the total intravenous-fluid volume in critically ill patients [2, 4]. The current study also demonstrated that fluid creep and maintenance fluid were the main sources of intravenous fluid intake, and the amount of fluid creep was similar to the results from the previous studies [2–4]. In addition, the amount of fluid creep tended to decrease in patients without hypoxemia and in those with mild hypoxemia, but not in those with severe hypoxemia. Although fluid restriction has been a common strategy in these patients [18, 19], conservative fluid management did not include fluid creep restriction [9–19]. Severe hypoxemia (P/F ≤ 150) is considered a threshold for more intensive management, including deep sedation, neuromuscular blockade use, and prone position [23–26]. These more intensive approaches, in addition to the severity of illness, may contribute to the increased fluid creep, including a higher dose of sedatives, analgesics, and vasopressors. Furthermore, patients with severe hypoxemia might undergo a longer period of mechanical ventilation [35]. Our findings imply that fluid creep should never be overlooked as an important source of intravenous-fluid volume, especially in patients with severe hypoxemia.

Sodium intake was reported to be 83–238 mEq/day in critically ill patients [20]. Notably, fluid creep is the major source of sodium intake in mechanically ventilated patients, and a higher sodium intake potentially contributes to hypernatremia and poor outcomes [7]. Chloride is also a common electrolyte for infusions; however, hyperchloremia has been reported to be a risk factor for acute kidney injury [8]. These huge burdens were observed in critically ill patients with mechanical ventilation, possibly contributing to edema development [21]. In our study, the mean intravenous sodium and chloride intake was approximately 300 mEq/day within 24 h of ICU admission, and 25% of both intakes were caused by fluid creep. After 24 h, the daily intake of sodium and chloride gradually decreased, whereas the proportion of the burdens due to fluid creep increased. Furthermore, larger amounts of sodium and chloride burdens were observed in patients with severe hypoxemia, and sodium burden did not decrease in patients with severe hypoxemia. Reducing sodium and chloride burdens may prevent organ dysfunction and improve outcomes in those patients.

Several randomized controlled trials comparing balanced crystalloids with normal saline demonstrated that the use of balanced crystalloids reduced hyperchloremia and major adverse kidney events, which was a composite of death, new renal replacement therapy, or persistent renal dysfunction [36, 37]. Furthermore, using not only resuscitative but also maintenance fluid with lower chloride levels was effective in significantly alleviating the chloride burden [16]. Compared with normal saline, usage of 5% dextrose as a drug diluent may reduce the risks of hypernatremia and hyperchloremia, without a higher risk of hyperglycemia [38]. Our study demonstrated that fluid creep was predominantly crystalloids that contain sodium and chloride. Because normal-saline use is rarely mandatory as a drug vehicle, the use of 5% dextrose as an alternative might contribute to reducing chloride load.

Resuscitation fluid restriction has been assessed in septic shock patients [9–15]. Although fluid restriction was feasible for the first several days, whether to reduce cumulative fluid balance was unclear in most of the previous studies. According to the international guidelines for the management of sepsis and septic shock [39], the insufficient evidence for outcome improvement could not provide conclusive recommendations for fluid restriction within 24 h after initial resuscitation. A recent randomized controlled trial [15] showed that most fluids were given outside the volumes specified by the fluid restriction protocol. Thus, fluid creep may weaken the effect of fluid restriction protocol. After adjustment of prespecified variables using a multiple linear regression model, our study demonstrated that hypoxemia was associated with a significant reduction in the total fluid intake but not with fluid creep, while septic shock increased either fluid volume. These results imply that fluid creep reduction is difficult to achieve even with the application of fluid restriction protocol. Considering the burden of fluid creep, total fluid management, including the resuscitation and non-resuscitation fluid, should be titrated for the reduction in cumulative fluid balance. Interventional studies are warranted to assess the feasibility of further fluid restriction in those patients.

### Strengths and limitations

To the best of our knowledge, no studies have assessed fluid creep according to oxygenation in mechanically ventilated adult patients. Our study suggests that fluid creep is a major source of fluid volume, sodium, and chloride in these patients. Furthermore, the amount of fluid creep did not decrease during the 7 days of ICU admission in patients with severe hypoxemia. Fluid creep may not be restricted enough even in those patients who commonly receive conservative fluid management.

This study has certain limitations. First, enteral intake data were not collected. However, enteral intake may be difficult to reduce for sufficient nutrition therapy. Our study provides adequate information to form a basis for further investigation of fluid restriction. Second, output data were not collected. Fluid balance is important in fluid therapy. However, considering the few differences in patient severity, mortality, and intravenous-fluid volume compared with previous studies [2, 3], the fluid balance might not have differed. Third, fluid management depends on local practice [3, 4]. As the study was exclusively conducted in Japan, our findings may have limited generalizability to other countries. Fourth, it is difficult to determine the reasons underlying fluid-therapy use retrospectively. We defined resuscitative fluid as isotonic crystalloid administered at > 166 mL/h, following a previous study [2]. Meanwhile, the definitions of maintenance fluid and fluid for catheter-patency maintenance were unclear in previous studies. Standardized definitions and classifications are required to further investigate the epidemiology of intravenous fluid therapy. Finally, we assessed the association between hypoxemia and the amount of fluid. However, we performed a multivariate analysis of intravenous fluid intake within 24 h, since data on oxygenation after 24 h were not collected. Further investigation based on daily oxygenation is warranted to confirm whether this association is consequently observed during the whole ICU stay.

## Conclusions

Fluid creep was the major source of fluid volume, sodium, and chloride among patients who underwent respiratory support, and the burdens due to fluid creep were prolonged in those with severe hypoxemia. Hypoxemia was associated with a significant reduction in the total fluid intake, but not with fluid creep. Since fluid creep was predominantly crystalloids containing sodium and chloride, these burdens can be decreased by the use of 5% dextrose. Interventional studies are required to assess the feasibility of further fluid restriction, including fluid creep, in these patients.

## Supplementary Information

**Additional file 1: Table S1.** Daily intravenous fluid intake according to fluid type. **Table S2.** Daily sodium intake according to fluid type. **Table S3.** Daily chloride intake according to fluid type. **Table S4.** Estimates of the effects of covariates on the fluid volume, sodium, and chloride in the multiple linear regression models.

## Data Availability

The data sets generated during and/or analyzed during the current study are not publicly available due to other post-hoc analyses by the co-authors but are available from the corresponding author on reasonable request.

## Abbreviations

APACHE-II: Acute physiology and chronic health evaluation II
CI: Confidence interval
ICU: Intensive care unit
IQR: Interquartile ranges
P/F ratio: Ratio of arterial oxygen partial pressure to fractional inspired oxygen
SD: Standard deviation
SOFA: Sequential organ failure assessment
