# Supplementary material for: A burden of fluid, sodium, and chloride due to intravenous fluid therapy in patients with respiratory support: a post-hoc analysis of a multicenter cohort study
Source: Ann Intensive Care. 2022 Oct 22;12:100. doi: 10.1186/s13613-022-01073-x (PMC9588139; doi:10.1186/s13613-022-01073-x)
Supplement: Supplementary file 1 — Additional file 1: Table S1. Daily intravenous fluid intake according to fluid type. Table S2. Daily sodium intake according to fluid type. Table S3. Daily chloride intake according to fluid type. Table S4. Estimates of the effects of covariates on the fluid volume, sodium, and chloride in the multiple linear regression models. [file 13613_2022_1073_MOESM1_ESM.docx]

**A burden of fluid, sodium, and chloride due to intravenous fluid therapy in mechanically ventilated patients: a post-hoc analysis of a multicenter cohort study**

**Supplementary results**

**Table of contents**

**Tables**

**Table S1** Daily intravenous fluid intake according to fluid type

………………………. 2-5

**Table S2** Daily sodium intake according to fluid type

………………………. 6-9

**Table S3** Daily chloride intake according to fluid type

……………………. 10-13

**Table S4** Estimates of the effects of covariates on the fluid volume, sodium, and chloride in the multiple linear regression models

……………………. 14-16

**Table S1** Daily intravenous fluid intake according to fluid type

1. All patients

| ICU stay | No. of patients | Total fluid, mL | Resuscitation | Blood product, mL | Colloid, mL | Maintenance | | Nutrition, mL | Fluid creep | | | | |
| --- | --- | --- | --- | --- | --- | --- | --- | --- | --- | --- | --- | --- | --- |
|  |  |  | Isotonic crystalloid, mL |  |  | Isotonic crystalloid, mL | Hypotonic crystalloid, mL |  | Antibiotics, mL | Sedation / analgesia, mL | Vasoactive drug, mL | Miscellaneous use, mL |  |
| –24 h | N=588 | 2662 (1646–4059)  3153 ± 2381 | 0 (0–500)  530 ± 1220 | 0 (0–189)  277 ± 960 | 0 (0–0)  130 ± 370 | 566 (0–1192)  742 ± 865 | 0 (0–291)  340 ± 697 | 0 (0–0)  117 ± 396 | 177 (50–300)  207 ± 209 | 116 (49–214)  160 ± 170 | 0 (0–102)  77 ± 158 | 176 (38.6–494)  351 ± 462 |  |
| 24–48 h | N=469 | 1739 (989–2794)  2171 ± 1840 | 0 (0–0)  121 ± 584 | 0 (0–0)  96 ± 423 | 0 (0–0)  45 ± 144 | 0 (0–571)  399 ± 728 | 0 (0–128)  290 ± 598 | 0 (0–0)  221 ± 727 | 200 (60–300)  220 ± 240 | 96 (25–199)  146 ± 198 | 0 (0–75)  61 ± 115 | 206 (23–523)  428 ± 734 |  |
| 48­–72 h | N=377 | 1477 (829–2595)  1938 ± 1772 | 0 (0–0)  63 ± 448 | 0 (0–0)  68 ± 373 | 0 (0–0)  47 ± 174 | 0 (0–0)  193 ± 489 | 0 (0–220)  305 ± 608 | 0 (0–0)  292 ± 744 | 150 (45–300)  225 ± 277 | 72 (0­–192)  141 ± 257 | 0 (0–43)  41 ± 92 | 212 (33–494)  406 ± 683 |  |
| 72–96 h | N=318 | 1384 (661–2314)  1752 ± 1513 | 0 (0–0)  35 ± 208 | 0 (0–0)  53 ± 300 | 0 (0–0)  26 ± 100 | 0 (0–0)  133 ± 394 | 0 (0–0)  276 ± 562 | 0 (0–48)  323 ± 818 | 150 (40–300)  204 ± 229 | 66 (0­–185)  116 ± 147 | 0 (0­–39)  38 ± 106 | 207 (25–480)  363 ± 598 |  |
| 96–120 h | N=265 | 1352 (596–2076)  1764 ± 1719 | 0 (0–0)  57 ± 318 | 0 (0–0)  54 ± 356 | 0 (0–0)  38 ± 155 | 0 (0–0)  100 ± 349 | 0 (0–0)  253 ± 585 | 0 (0–30)  403 ± 1085 | 100 (0–250)  186 ± 232 | 68 (0–160)  117 ± 159 | 0 (0­–24)  36 ± 138 | 192 (15–477)  365 ± 644 |  |
| 120–144 h | N=200 | 1310 (527–2145)  1716 ± 1750 | 0 (0–0)  17 ± 96 | 0 (0–0)  65 ± 380 | 0 (0–0)  35 ± 137 | 0 (0–0)  66 ± 267 | 0 (0–0)  201 ± 582 | 0 (0–465)  525 ± 1395 | 100 (0–200)  170 ± 255 | 66 (0–162)  114 ± 147 | 0 (0­–31)  31 ± 61 | 192 (12–480)  329 ± 464 |  |
| 144–168 h | N=170 | 1250 (572–2080)  1683 ± 1832 | 0 (0–0)  16 ± 112 | 0 (0–0)  72 ± 497 | 0 (0–0)  38 ± 128 | 0 (0–0)  110 ± 360 | 0 (0–0)  160 ± 420 | 0 (0–423)  518 ± 1375 | 100 (0–250)  172 ± 220 | 67 (0–169)  114 ± 150 | 0 (0­–24)  33 ± 79 | 171 (0–480)  326 ± 493 |  |

The amount of each fluid was presented as a median (interquartile range) and mean ± standard deviation.

Miscellaneous fluid included vehicles for any other drugs and continuous infusions at low rate (≤ 20 mL/h).

Abbreviations: ICU, intensive care unit.

1. Patients without hypoxemia (P/F > 300)

| ICU stay | No. of patients | Total fluid, mL | Resuscitation | Blood product, mL | Colloid, mL | Maintenance | | Nutrition, mL | Fluid creep | | | |
| --- | --- | --- | --- | --- | --- | --- | --- | --- | --- | --- | --- | --- |
|  |  |  | Isotonic crystalloid, mL |  |  | Isotonic crystalloid, mL | Hypotonic crystalloid, mL |  | Antibiotics, mL | Sedation / analgesia, mL | Vasoactive drug, mL | Miscellaneous use, mL |
| –24 h | N=167 | 2924 (1796–4043)  3331 ± 2395 | 0 (0–500)  599 ± 1410) | 0 (0–0)  182 ± 449 | 0 (0–0)  142 ± 521 | 789 (0–1223)  834 ± 891 | 0 (0–800)  498 ± 903 | 0 (0–0)  76 ± 256 | 200 (60–300)  197 ± 181 | 158 (70–275)  201 ± 188 | 0 (0–36)  61 ± 204 | 104 (0–470)  312 ± 506 |
| 24–48 h | N=111 | 1660 (1148–2869)  2295 ± 2053 | 0 (0–0)  141 ± 680) | 0 (0–0)  74 ± 279 | 0 (0–0)  60 ± 174 | 0 (0–783)  449 ± 784 | 0 (0–393)  340 ± 659 | 0 (0–0)  185 ± 500 | 200 (100–300)  216 ± 172 | 89 (15–213)  173 ± 304 | 0 (0–24)  33 ± 84 | 100 (0–485)  443 ± 973 |
| 48­–72 h | N=75 | 1653 (913–2719)  2031 ± 1844 | 0 (0–0)  44 ± 379) | 0 (0–0)  22 ± 97 | 0 (0–0)  50 ± 197 | 0 (0–95)  248 ± 509 | 0 (0–500)  337 ± 602 | 0 (0–143)  332 ± 779 | 200 (50–300)  256 ± 284 | 30 (0–142)  142 ± 313 | 0 (0–26)  24 ± 54 | 198 (10–480)  437 ± 910 |
| 72–96 h | N=59 | 1508 (712–2484)  1999 ± 1845 | 0 (0–0)  53 ± 286 | 0 (0–0)  33 ±141 | 0 (0–0)  25 ± 104 | 0 (0–223)  250 ± 520 | 0 (0–0)  286 ± 552 | 0 (0–480)  425 ± 905 | 150 (40–250)  184 ± 188 | 18 (0–120)  89 ± 155 | 0 (0–0)  21 ± 52 | 240 (10–480)  467 ± 967 |
| 96–120 h | N=45 | 1676 (741–2104)  1944 ± 1931 | 0 (0–0)  35 ± 171 | 0 (0–0)  21 ± 100 | 0 (0–0)  22 ± 97 | 0 (0–0)  210 ± 462 | 0 (0–0)  214 ± 418 | 0 (0–480)  450 ± 1270 | 150 (0–200)  172 ± 287 | 28 (0–105)  118 ± 215 | 0 (0–0)  22 ± 64 | 240 (0–527)  536 ± 1093 |
| 120–144 h | N=34 | 1313 (480–2136)  1618 ± 1729 | 0 (0–0)  12 ± 71 | 0 (0–0)  0 ± 0 | 0 (0–0)  17 ± 69 | 0 (0–0)  192 ± 498 | 0 (0–0)  154 ± 352 | 0 (0–480)  572 ± 1510 | 74 (0–150)  140 ± 267 | 12 (0–150)  87 ± 144 | 0 (0–0)  12 ± 29 | 191 (0–552)  325 ± 397 |
| 144–168 h | N=25 | 1297 (313–1956)  1548 ± 1568 | 0 (0–0)  20 ± 100 | 0 (0–0)  0 ± 0 | 0 (0–0)  47 ± 136 | 0 (0–0)  299 ± 595 | 0 (0–457)  298 ± 583 | 0 (0–14)  397 ± 1145 | 0 (0–133)  83 ± 124 | 13 (0–100)  57 ± 76 | 0 (0–0)  16 ± 39 | 97 (0–313)  223 ± 323 |

The amount of each fluid was presented as a median (interquartile range) and mean ± standard deviation.

Abbreviations: ICU, intensive care unit; KVO, keep venous open.

1. Patients with mild hypoxemia (150 < P/F ≤ 300)

| ICU stay | No. of patients | Total fluid, mL | Resuscitation | Blood product, mL | Colloid, mL | Maintenance | | Nutrition, mL | Fluid creep | | | |
| --- | --- | --- | --- | --- | --- | --- | --- | --- | --- | --- | --- | --- |
|  |  |  | Isotonic crystalloid, mL |  |  | Isotonic crystalloid, mL | Hypotonic crystalloid, mL |  | Antibiotics, mL | Sedation / analgesia, mL | Vasoactive drug, mL | Miscellaneous use, mL |
| –24 h | N=297 | 2617 (1517–3999)  3058 ± 2395 | 0 (0–500)  554 ± 1179 | 0 (0–280)  288 ± 976 | 0 (0–46)  114 ± 253 | 504 (0–1127)  795 ± 841 | 0 (0–0)  268 ± 580 | 0 (0–0)  120 ± 424 | 150 (50–300)  195 ± 197 | 99 (44­–177)  139 ± 151 | 11 (0–111)  79 ± 138 | 196 (48–548)  368 ± 456 |
| 24–48 h | N=247 | 1606 (926–2696)  2084 ± 1856 | 0 (0–0)  137 ± 636 | 0 (0–0)  83 ± 363 | 0 (0–0)  38 ± 136 | 0 (0–530)  365 ± 647 | 0 (0–0)  253 ± 568 | 0 (0–0)  231 ± 855 | 150 (60–300)  205 ± 212 | 93 (25–166)  124 ± 135 | 0 (0–90)  65 ± 119 | 217 (40–538)  428 ± 693 |
| 48­–72 h | N=199 | 1425 (761–2350)  1891 ± 1915 | 0 (0–0)  74 ± 528 | 0 (0–0)  96 ± 471 | 0 (0–0)  55 ± 197 | 0 (0–0)  168 ± 399 | 0 (0–0)  272 ± 605 | 0 (0–0)  280 ± 745 | 150 (41–300)  217 ± 292 | 69 (0–169)  127 ± 272 | 0 (0–43)  40 ± 84 | 219 (33–513)  403 ± 657 |
| 72–96 h | N=168 | 1243 (543–2117)  1600 ± 1472 | 0 (0–0)  33 ± 194 | 0 (0–0)  65 ± 376 | 0 (0–0)  34 ± 119 | 0 (0­–0)  85 ± 309 | 0 (0–0)  252 ± 562 | 0 (0–0)  297 ± 773 | 106 (40–285)  194 ± 225 | 70 (0–176)  110 ± 133 | 0 (0–44)  34 ± 70 | 202 (23–480)  321 ± 457 |
| 96–120 h | N=141 | 1130 (501–2060)  1640 ± 1687 | 0 (0–0)  75 ± 405 | 0 (0–0)  75 ± 474 | 0 (0–0)  51 ± 197 | 0 (0–0)  84 ± 343 | 0 (0–0)  195 ± 460 | 0 (0–30)  382 ± 996 | 100 (0–250)  189 ± 237 | 69 (0–144)  100 ± 122 | 0 (0–24)  45 ± 180 | 160 (8–401)  304 ± 501 |
| 120–144 h | N=108 | 1000 (503–2132)  1642 ± 1768 | 0 (0–0)  21 ± 114 | 0 (0–0)  110 ± 509 | 0 (0–0)  45 ± 152 | 0 (0–0)  49 ± 198 | 0 (0–0)  153 ± 440 | 0 (0–228)  496 ± 132) | 100 (5–232)  174 ± 279 | 68 (5–137)  102 ± 127 | 0 (0–30)  31 ± 58 | 143 (–378)  297 ± 520 |
| 144–168 h | N=98 | 1147 (594–2102)  1710 ± 2045 | 0 (0–0)  22 ± 139 | 0 (0–0)  115 ± 651 | 0 (0–0)  35 ± 135 | 0 (0–0)  99 ± 339 | 0 (0–0)  116 ± 341 | 0 (0–470)  576 ± 1547 | 100 (40–250)  161 ± 173 | 61 (0–151)  105 ± 135 | 0 (0–24)  31 ± 66 | 155 (10–393)  304 ± 527 |

The amount of each fluid was presented as a median (interquartile range) and mean ± standard deviation.

Abbreviations: ICU, intensive care unit; KVO, keep venous open.

1. Patients with severe hypoxemia (P/F ≤ 150)

| ICU stay | No. of patients | Total fluid, mL | Resuscitation | Blood product, mL | Colloid, mL | Maintenance | | Nutrition, mL | Fluid creep | | | |
| --- | --- | --- | --- | --- | --- | --- | --- | --- | --- | --- | --- | --- |
|  |  |  | Isotonic crystalloid, mL |  |  | Isotonic crystalloid, mL | Hypotonic crystalloid, mL |  | Antibiotics, mL | Sedation / analgesia, mL | Vasoactive drug, mL | Miscellaneous use, mL |
| –24 h | N=124 | 2712 (1649–4215)  3210 (2362) | 0 (0–500)  383 ± 1019 | 0 (0–0)  380 ± 1346 | 0 (0–75)  165 ± 391 | 110 (0–1255)  706 ± 887) | 0 (0–363)  301 ± 600 | 0 (0–0)  168 ± 470 | 200 (55–350)  250 ± 262 | 106 (38–200)  155 ± 175 | 27 (0–161)  92 ± 127 | 242 (45–496)  362 ± 414 |
| 24–48 h | N=111 | 1819 (1093–3162)  2294 (1626) | 0 (0–0)  66 ± 286 | 0 (0–0)  147 ± 620 | 0 (0–0)  50 ± 139 | 0 (0–572)  428 ± 836 | 0 (0–383)  324 ± 598 | 0 (0–30)  237 ± 601 | 200 (50–300)  258 ± 337 | 123 (35–244)  167 ± 178 | 8 (0–1100  78 ± 128 | 257 (40–541)  414 ± 520 |
| 48­–72 h | N=103 | 1515 (872–2723)  1941 (1365) | 0 (0–0)  57 ± 307 | 0 (0–0)  47 ± 269 | 0 (0–0)  33 ± 92 | 0 (0–0)  203 ± 617 | 0 (0–660)  344 ± 619 | 0 (0–177)  284 ± 721 | 200 (50–300)  256 ± 284 | 122 (44–235)  165 ± 165 | 0 (0–74)  55 ± 122 | 213 (42–500)  389 ± 527 |
| 72–96 h | N=91 | 1388 (851–2365)  1826 (1296) | 0 (0–0)  29 ± 173 | 0 (0–0)  42 ± 201 | 0 (0–0)  10 ± 28 | 0 (0–0)  145 ± 428 | 0 (0–437)  316 ± 573 | 0 (0–0)  305 ± 844 | 200 (50–300)  238 ± 257 | 93 (16–226)  145 ± 163 | 0 (0–67)  56 ± 168 | 200 (44–480)  373 ± 509 |
| 96–120 h | N=79 | 1404 (688–2162)  1807 (1629) | 0 (0–0)  37 ± 173 | 0 (0–0)  35 ± 133 | 0 (0–0)  22 ± 67 | 0 (0–0)  67 ± 266 | 0 (0–394)  378 ± 809 | 0 (0–0)  414 ± 1138 | 150 (0–300)  188 ± 188 | 97 (0–206)  146 ± 178 | 0 (0–37)  28 ± 62 | 192 (38–510)  376 ± 505 |
| 120–144 h | N=58 | 1398 (712–2307)  1833 (1737) | 0 (0–0)  10 ± 66 | 0 (0–0)  18 ± 92 | 0 (0–0)  26 ± 133 | 0 (0–0)  22 ± 144 | 0 (0–0)  317 ± 853 | 0 (0–677)  552 ± 1476 | 117 (0–250)  179 ± 195 | 97 (12–239)  150 ± 175 | 0 (0–54)  41 ± 77 | 261 (50–606)  391 ± 383 |
| 144–168 h | N=47 | 1334 (572–2080)  1697 (1490) | 0 (0–0)  0 ± 0 | 0 (0–0)  20 ± 75 | 0 (0–0)  40 ± 109 | 0 (0–0)  30 ± 150 | 0 (0–0)  177 ± 460 | 0 (0–295)  462 ± 1096 | 177 (20–300)  243 ± 311 | 122 (20–221)  163 ± 191 | 0 (0–82)  48 ± 111 | 352 (13–590)  424 ± 487 |

The amount of each fluid was presented as a median (interquartile range) and mean ± standard deviation.

Abbreviations: ICU, intensive care unit; KVO, keep venous open.

**Table S2** Daily sodium intake according to fluid type

1. All patients

| ICU stay | No. of patients | Total fluid, mEq | Resuscitation | Blood product, mEq | Colloid, mEq | Maintenance | | Nutrition, mEq | Fluid creep | | | |
| --- | --- | --- | --- | --- | --- | --- | --- | --- | --- | --- | --- | --- |
|  |  |  | Isotonic crystalloid, mEq |  |  | Isotonic crystalloid, mEq | Hypotonic crystalloid, mEq |  | Antibiotics, mEq | Sedation / analgesia, mEq | Vasoactive drug, mEq | Miscellaneous use, mEq |
| –24 h | N=588 | 261.2 (144.6–420.5)  335.1 ± 315.0 | 0 (0–65.5)  69.9 ± 161.2 | 0 (0–15.7)  37.1 ± 131.8 | 0 (0–0)  16.0 ± 48.2 | 74.6 (0–155.1)  97.5 ± 113.8 | 0 (0–0)  14.3 ± 33.8 | 0 (0–0)  2.6 ± 13.8 | 20.0 (0–46.2)  28.4 ± 63.5 | 5.9 (0–14.1)  8.8 ± 10.3 | 0 (0–8.9)  7.0 ± 14.4 | 10.5 (0–41.4)  26.5 ± 36.6 |
| 24–48 h | N=469 | 152.2 (79.4–250.9)  197.7 ± 190.0 | 0 (0–0)  15.8 ± 76.3 | 0 (0–0)  12.6 ± 57.2 | 0 (0–0)  4.8 ± 18.7 | 0 (0–74.3)  52.6 ± 96.0 | 0 (0–0)  13.9 ± 34.5 | 0 (0–0)  7.2 ± 30.3 | 23.1 (3.1–46.2)  26.8 ± 31.7 | 5.3 (0–14.8)  10.3 ± 20.8 | 0 (0–7.9)  6.2 ± 13.1 | 12.3 (0–47.1)  32.8 ± 49.7 |
| 48­–72 h | N=377 | 117.1 (64.5–212.5)  161.7 ± 168.5 | 0 (0–0)  8.3 ± 58.5 | 0 (0–0)  9.2 ± 53.5 | 0 (0–0)  4.6 ± 20.8 | 0 (0–0)  25.3 ± 64.1 | 0 (0–0)  15.4 ± 37.6 | 0 (0–0)  11.6 ± 35.0 | 15.4 (0–46.2)  27.3 ± 37.1 | 5.6 (0–14.8)  10.3 ± 21.3 | 0 (0–3.4)  4.6 ± 10.6 | 11.9 (0–42.3)  28.5 ± 41.9 |
| 72–96 h | N=318 | 96.5 (48.6–166.8)  133.5 ± 133.1 | 0 (0–0)  4.7 ± 27.2 | 0 (0–0)  7.2 ± 42.6 | 0 (0–0)  2.4 ± 12.5 | 0 (0–0)  17.6 ± 52.0 | 0 (0–0)  12.4 ± 30.8 | 0 (0–0)  12.9 ± 37.8 | 15.4 (0–38.5)  23.9 ± 29.3 | 0 (0–0)  0.2 ± 2.1 | 0 (0–0.1)  3.6 ± 8.5 | 6.2 (0–33.6)  22.3 ± 36.1 |
| 96–120 h | N=265 | 90.3 (42.0–160.2)  126.4 ± 139.7 | 0 (0–0)  7.5 ± 41.6 | 0 (0–0)  7.8 ± 53.0 | 0 (0–0)  3.3 ± 15.4 | 0 (0–0)  13.3 ± 46.5 | 0 (0–0)  0 ± 0 | 0 (0–0)  15.4 ± 47.9 | 12.3 (0–30.8)  21.3 ± 28.3 | 3.7 (0–13.4)  8.7 ± 12.4 | 0 (0–0.1)  4.3 ± 20.4 | 7.8 (0–29.8)  22.7 ± 37.6 |
| 120–144 h | N=200 | 82.0 (40.0–153.7)  115.3 ± 123.8 | 0 (0–0)  2.2 ± 12.6 | 0 (0–0)  8.8 ± 53.9 | 0 (0–0)  3.0 ± 15.5 | 0 (0–0)  8.8 ± 36.6 | 0 (0–0)  6.5 ± 21.4 | 0 (0–0.8)  18.8 ± 51.4 | 9.8 (0–30.8)  19.4 ± 26.1 | 3.8 (0–14.8)  9.2 ± 13.0 | 0 (0–1.3)  3.1 ± 6.9 | 9.1 (0–33.6)  23.2 ± 33.5 |
| 144–168 h | N=170 | 84.0 (39.9–160.8)  119.3 ± 135.1 | 0 (0–0)  2.2 ± 15.9 | 0 (0–0)  10.0 ± 71.3 | 0 (0–0)  3.9 ± 16.5 | 0 (0–0)  14.9 ± 49.1 | 0 (0–0)  5.0 ± 14.3 | 0 (0–0)  19.3 ± 52.0 | 9.2 (0–30.8)  21.1 ± 31.9 | 2.9 (0–14.8)  9.5 ± 14.1 | 0 (0–0)  3.1 ± 8.3 | 16.8 (0–60.5)  52.3 ± 96.3 |

The amount of each fluid was presented as a median (interquartile range) and mean ± standard deviation.

Abbreviations: ICU, intensive care unit; KVO, keep venous open.

1. Patients without hypoxemia (P/F > 300)

| ICU stay | No. of patients | Total fluid, mEq | Resuscitation | Blood product, mEq | Colloid, mEq | Maintenance | | Nutrition, mEq | Fluid creep | | | |
| --- | --- | --- | --- | --- | --- | --- | --- | --- | --- | --- | --- | --- |
|  |  |  | Isotonic crystalloid, mEq |  |  | Isotonic crystalloid, mEq | Hypotonic crystalloid, mEq |  | Antibiotics, mEq | Sedation / analgesia, mEq | Vasoactive drug, mEq | Miscellaneous use, mEq |
| –24 h | N=167 | 265.6 (160.8–455.1)  349.1 ± 316.1 | 0 (0–65.5)  79.4 ± 189.2 | 0 (0–0)  24.1 ± 61.0 | 0 (0–0)  18.0 ± 67.8 | 108.1 (0–160.2)  109.5 ± 116.6 | 0 (0–26.8)  20.5 ± 40.3 | 0 (0­-0)  2.1 ± 9.9 | 30.8 (0–46.2)  26.8 ± 25.9 | 5.7 (0–13.4)  9.4 ± 11.5 | 0 (0–1.4)  3.4 ± 11.1 | 7.7 (0–45.3)  24.8 ± 35.2 |
| 24–48 h | N=111 | 166.5 (96.3–250.9)  209.9 ± 196.0 | 0 (0–0)  18.4 ± 88.4 | 0 (0–0)  8.8 ± 35.2 | 0 (0–0)  7.1 ± 22.4 | 0 (0–102.2)  58.9 ± 102.7 | 0 (0–13.8)  15.1 ± 32.3 | 0 (0­-0)  7.0 ± 22.3 | 30.8 (0–46.2)  28.5 ± 25.1 | 4.1 (0–14.1)  12.2 ± 37.4 | 0 (0–0.3)  3.0 ± 9.1 | 7.7 (0–56.3)  35.9 ± 61.6 |
| 48­–72 h | N=75 | 133.5 (72.0–213.4)  163.8 ± 144.1 | 0 (0–0)  5.7 ± 49.3 | 0 (0–0)  2.4 ± 10.7 | 0 (0–0)  4.9 ± 24.7 | 0 (0–12.4)  32.7 ± 67.5 | 0 (0–6.2)  14.7 ± 30.8 | 0 (0­-0)  13.7 ± 36.2 | 15.4 (0–46.2)  26.0 ± 29.2 | 1.4 (0–11.9)  11.3 ± 39.9 | 0 (0–0)  2.2 ± 7.1 | 9.4 (0–55.4)  30.9 ± 52.0 |
| 72–96 h | N=59 | 137.7 (62.7–211.3)  156.0 ± 129.4 | 0 (0–0)  6.9 ± 37.2 | 0 (0–0)  4.2 ± 19.0 | 0 (0–0)  2.4 ± 13.1 | 0 (0–28.9)  33.5 ± 69.7 | 0 (0–0)  13.4 ± 28.0 | 0 (0­-4.5)  18.2 ± 43.9 | 15.4 (0–32.9)  22.5 ± 24.9 | 0 (0–0)  0.1 ± 0.8 | 0 (0–0)  2.0 ± 6.7 | 7.4 (0–44.9)  29.7 ± 49.6 |
| 96–120 h | N=45 | 115.3 (64.8–176.7)  146.1 ± 122.8 | 0 (0–0)  4.6 ± 22.3 | 0 (0–0)  2.8 ± 13.2 | 0 (0–0)  2.5 ± 12.5 | 0 (0–0)  28.4 ± 64.4 | 0 (0–0)  0 ± 0 | 0 (0­-16.8)  20.9 ± 63.1 | 12.9 (0–30.8)  22.9 ± 38.9 | 0 (0–7.4)  6.3 ± 11.3 | 0 (0–0)  2.7 ± 9.5 | 23.4 (0–40.8)  33.7 ± 55.2 |
| 120–144 h | N=34 | 93.3 (43.5–164.4)  121.9 ± 116.0 | 0 (0–0)  1.6 ± 9.4 | 0 (0–0)  0 ± 0 | 0 (0–0)  1.8 ± 10.3 | 0 (0–0)  26.4 ± 69.9 | 0 (0­–0)  5.4 ± 12.8 | 0 (0­-16.8)  27.6 ± 75.5 | 3.1 (0–23.1)  17.9 ± 33.0 | 0 (0–8.2)  7.2 ± 12.1 | 0 (0–0)  1.2 ± 3.5 | 7.1 (0–33.6)  19.7 ± 24.4 |
| 144–168 h | N=25 | 108.8 (17.8–156.8)  117.3 ± 107.4 | 0 (0–0)  2.6 ± 13.0 | 0 (0–0)  0 ± 0 | 0 (0–0)  6.0 ± 20.9 | 0 (0–51.8)  41.4 ± 83.2 | 0 (0–0)  7.1 ± 13.5 | 0 (0­-0)  18.4 ± 56.0 | 0 (0–10.0)  9.9 ± 18.1 | 0 (0–7.4)  4.8 ± 8.6 | 0 (0–0)  1.1 ± 3.9 | 1.6 (0–31.4)  16.3 ± 30.4 |

The amount of each fluid was presented as a median (interquartile range) and mean ± standard deviation.

Abbreviations: ICU, intensive care unit; KVO, keep venous open.

1. Patients with mild hypoxemia (150 < P/F ≤ 300)

| ICU stay | No. of patients | Total fluid, mEq | Resuscitation | Blood product, mEq | Colloid, mEq | Maintenance | | Nutrition, mEq | Fluid creep | | | |
| --- | --- | --- | --- | --- | --- | --- | --- | --- | --- | --- | --- | --- |
|  |  |  | Isotonic crystalloid, mEq |  |  | Isotonic crystalloid, mEq | Hypotonic crystalloid, mEq |  | Antibiotics, mEq | Sedation / analgesia, mEq | Vasoactive drug, mEq | Miscellaneous use, mEq |
| –24 h | N=297 | 257.1 (140.2–407.3)  323.8 ± 314.2 | 0 (0–65.5)  72.8 ± 154.6 | 0 (0­–30.8)  38.4 ± 129.7 | 0 (0–0)  12.8 ± 29.4 | 65.5 (0–148.3)  92.9 ± 111.0 | 0 (0–0)  10.3 ± 27.6 | 0 (0–0)  2.2 ± 12.7 | 15.4 (0–37.2)  28.1 ± 83.9 | 5.5 (13.4)  7.8 ± 8.9 | 0 (0–11.1)  7.8 ± 15.1 | 9.2 (0–40.6)  26.6 ± 37.8 |
| 24–48 h | N=247 | 137.3 (75.3–228.8)  187.5 ± 189.5 | 0 (0–0)  18.0 ± 83.1 | 0 (0–0)  11.5 ± 52.2 | 0 (0–0)  3.7 ± 17.2 | 0 (0–69.4)  48.2 ± 86.3 | 0 (0–0)  11.5 ± 32.4 | 0 (0–0)  6.7 ± 33.7 | 17.2 (3.1–36.1)  24.7 ± 25.9 | 4.8 (0–14.8)  8.7 ± 11.0 | 0 (0–9.9)  7.0 ± 13.8 | 12.4 (0–44.6)  32.7 ± 49.4 |
| 48­–72 h | N=199 | 109.8 (56.5–204.0)  160.3 ± 190.8 | 0 (0–0)  9.7 ± 68.7 | 0 (0–0)  13.1 ± 68.1 | 0 (0–0)  5.0 ± 22.9 | 0 (0–0)  21.9 ± 52.4 | 0 (0–0)  14.2 ± 39.6 | 0 (0–0)  11.2 ± 34.8 | 15.4 (0–37.9)  26.0 ± 39.4 | 4.1 (0–13.5)  8.9 ± 12.6 | 0 (0–5.2)  4.7 ± 9.7 | 11.3 (0–41.8)  28.4 ± 41.9 |
| 72–96 h | N=168 | 80.9 (43.1–156.6)  126.5 ± 143.9 | 0 (0–0)  4.2 ± 25.3 | 0 (0–0)  9.4 ± 54.5 | 0 (0–0)  3.6 ± 15.3 | 0 (0–0)  11.1 ± 40.3 | 0 (0–0)  11.9 ± 33.4 | 0 (0–0)  12.2 ± 37.0 | 15.3 (0–30.8)  22.3 ± 27.4 | 0 (0–0)  0.0 ± 0.3 | 0 (0–2.5)  3.7 ± 7.5 | 5.0 (0–25.7)  19.0 ± 31.3 |
| 96–120 h | N=141 | 76.6 (32.0–146.9)  123.4 ± 162.0 | 0 (0–0)  9.9 ± 53.0 | 0 (0–0)  10.8 ± 70.7 | 0 (0–0)  4.5 ± 19.0 | 0 (0–0)  11.0 ± 44.7 | 0 (0–0)  0 ± 0 | 0 (0–0)  15.6 ± 48.1 | 9.2 (0–30.8)  19.2 ± 24.4 | 2.7 (0–13.4)  7.9 ± 10.9 | 0 (0–0.6)  5.1 ± 26.5 | 7.3 (0–20.4)  19.4 ± 34.5 |
| 120–144 h | N=108 | 74.1 (36.1–141.6)  114.4 ± 142.8 | 0 (0–0)  2.8 ± 15.0 | 0 (0–0)  15.1 ± 72.4 | 0 (0–0)  3.9 ± 16.1 | 0 (0–0)  6.5 ± 25.9 | 0 (0–0)  4.5 ± 15.0 | 0 (0–0)  18.3 ± 50.4 | 9.2 (0–25.8)  17.2 ± 21.4 | 3.3 (0–14.2)  8.3 ± 11.6 | 0 (0–2.1)  3.3 ± 6.7 | 7.6 (0–27.3)  22.3 ± 37.0 |
| 144–168 h | N=98 | 78.0 (35.0–144.4)  116.4 ± 153.9 | 0 (0–0)  3.1 ± 19.8 | 0 (0–0)  16.3 ± 93.4 | 0 (0–0)  4.0 ± 17.5 | 0 (0–0)  13.3 ± 45.3 | 0 (0–0)  3.5 ± 12.2 | 0 (0–10.2)  18.2 ± 47.0 | 9.2 (0–24.6)  17.6 ± 22.4 | 2.6 (0–12.1)  8.6 ± 12.8 | 0 (0–1.1)  3.3 ± 7.1 | 15.9 (0–69.3)  52.4 ± 90.0 |

The amount of each fluid was presented as a median (interquartile range) and mean ± standard deviation.

Abbreviations: ICU, intensive care unit; KVO, keep venous open.

1. Patients with severe hypoxemia (P/F ≤ 150)

| ICU stay | No. of patients | Total fluid, mEq | Resuscitation | Blood product, mEq | Colloid, mEq | Maintenance | | Nutrition, mEq | Fluid creep | | | |
| --- | --- | --- | --- | --- | --- | --- | --- | --- | --- | --- | --- | --- |
|  |  |  | Isotonic crystalloid, mEq |  |  | Isotonic crystalloid, mEq | Hypotonic crystalloid, mEq |  | Antibiotics, mEq | Sedation / analgesia, mEq | Vasoactive drug, mEq | Miscellaneous use, mEq |
| –24 h | N=124 | 267.1 (108.0–448.0)  343.2 ± 317.1 | 0 (0–65)  50.1 ± 132.8 | 0 (0­–0)  51.5 ± 192.3 | 0 (0–0)  20.8 ± 52.5 | 14.3 (0–163.7)  92.6 ± 116.4 | 0 (0–0)  15.4 ± 36.4 | 0 (0–0)  4.2 ± 19.7 | 15.4 (0–46.2)  31.5 ± 37.5 | 6.9 (0–15.5)  10.2 ± 11.4 | 0 (0–16.4)  10.1 ± 15.7 | 15.4 (3.1–41.5)  28.5 ± 35.6 |
| 24–48 h | N=111 | 164.6 (79.8–268.2)  208.0 ± 185.4 | 0 (0–0)  8.7 ± 37.8 | 0 (0­–0)  18.9 ± 80.7 | 0 (0–0)  5.2 ± 17.9 | 0 (0–74.3)  56.1 ± 109.3 | 0 (0–13.4)  18.1 ± 40.4 | 0 (0–0)  8.6 ± 29.2 | 15.4 (3.1–46.2)  29.8 ± 46.1 | 8.3 (0–16.7)  11.9 ± 13.0 | 0 (0­­–10.4)  7.8 ± 14.6 | 15.4 (2.3–48.1)  29.8 ± 34.8 |
| 48­–72 h | N=103 | 127.7 (69.6–227.8)  162.9 ± 137.4 | 0 (0–0)  7.6 ± 40.6 | 0 (0–0)  6.6 ± 37.4 | 0 (0–0)  3.4 ± 11.7 | 0 (0–0)  26.5 ± 80.3 | 0 (0–28.1)  18.1 ± 38.3 | 0 (0–0)  11.0 ± 34.) | 15.4 (0–46.2)  30.7 ± 37.7 | 9.2 (0–17.0)  12.5 ± 14.2 | 0 (0–4.7)  5.9 ± 13.8 | 13.5 (1.4–41.4)  27.0 ± 33.0 |
| 72–96 h | N=91 | 95.8 (51.5–174.9)  132.1 ± 112.8 | 0 (0–0)  3.9 ± 23.0 | 0 (0–0)  5.1 ± 24.9 | 0 (0–0)  0.0 ± 0.5 | 0 (0–0)  19.1 ± 56.0 | 0 (0–8.4)  12.9 ± 27.7 | 0 (0–0)  10.5 ± 34.9 | 15.4 (0–46.2)  27.7 ± 34.7 | 0 (0–0)  0.5 ± 3.9 | 0 (0–2.1)  4.7 ± 10.8 | 9.1 (0–27.7)  23.5 ± 33.5 |
| 96–120 h | N=79 | 92.8 (46.1–160.2)  120.3 ± 100.8 | 0 (0–0)  4.8 ± 22.4 | 0 (0–0)  5.3 ± 20.0 | 0 (0–0)  1.4 ± 7.4 | 0 (0–0)  8.8 ± 35.0 | 0 (0­–0)  0 ± 0 | 0 (0–0)  11.9 ± 36.5 | 15.4 (0–46.2)  24.1 ± 27.6 | 6.1 (0–14.8)  11.2 ± 15.0 | 0 (0–2.6)  3.9 ± 9.2 | 12.3 (0.8–29.8)  22.3 ± 28.8 |
| 120–144 h | N=58 | 87.6 (45.2–179.3)  113.1 ± 86.2 | 0 (0–0)  1.4 ± 9.0 | 0 (0–0)  2.0 ± 10.8 | 0 (0–0)  2.2 ± 17.1 | 0 (0–0)  2.9 ± 18.8 | 0 (0–0)  11.0 ± 32.5 | 0 (0–0)  14.6 ± 33.0 | 15.4 (0–34.7)  24.3 ± 29.2 | 7.4 (0–17.3)  11.9 ± 15.4 | 0 (0–4.8)  3.9 ± 8.3 | 17.1 (0.8–44.6)  26.8 ± 31.3 |
| 144–168 h | N=47 | 97.1 (51.1–197.8)  126.5 ± 104.9 | 0 (0–0)  0 ± 0 | 0 (0–0)  2.3 ± 8.7 | 0 (0–0)  2.6 ± 11.0 | 0 (0–0)  4.2 ± 20.6 | 0 (0–0)  7.0 ± 18.1 | 0 (0–0)  21.9 ± 60.1 | 23.1 (0–46.2)  34.3 ± 47.3 | 7.8 (0–20.3)  14.0 ± 17.6 | 0 (0­–0)  3.8 ± 11.7 | 31.5 (8.4–69.0)  71.3 ± 124.2 |

The amount of each fluid was presented as a median (interquartile range) and mean ± standard deviation.

Abbreviations: ICU, intensive care unit; KVO, keep venous open.

**Table S3** Daily chloride intake according to fluid type

1. All patients

| ICU stay | No. of patients | Total fluid, mEq | Resuscitation | Blood product, mEq | Colloid, mEq | Maintenance | | Nutrition, mEq | Fluid creep | | | |
| --- | --- | --- | --- | --- | --- | --- | --- | --- | --- | --- | --- | --- |
|  |  |  | Isotonic crystalloid, mEq |  |  | Isotonic crystalloid, mEq | Hypotonic crystalloid, mEq |  | Antibiotics, mEq | Sedation / analgesia, mEq | Vasoactive drug, mEq | Miscellaneous use, mEq |
| –24 h | N=588 | 235.2 (137.2–384.9)  298.2 ± 274.3 | 0 (0–54.7)  59.2 ± 137.6 | 0 (0–15.7)  37.1 ± 131.8 | 0 (0–0)  15.7 ± 45.2 | 62.7 (0–130.1)  82.2 ± 96.1 | 0 (0–0)  13.1 ± 29.8 | 0 (0–0)  2.3 ± 11.5 | 20.0 (0–46.2)  28.4 ± 63.5 | 5.9 (0–14.1)  8.8 ± 10.3 | 0 (0–8.9)  7.0 ± 14.4 | 12.3 (0–45.8)  28.7 ± 39.7 |
| 24–48 h | N=469 | 140.8 (78.3–224.9)  182.1 ± 173.0 | 0 (0–0)  13.4 ± 64.2 | 0 (0–0)  12.6 ± 57.2 | 0 (0–0)  4.7 ± 18.4 | 0 (0–62.3)  44.6 ± 81.7 | 0 (0–0)  12.5 ± 29.2 | 0 (0–0  6.6 ± 28.4 | 23.1 (3.1–46.2)  26.8 ± 31.7 | 5.3 (0–14.8)  10.3 ± 20.8 | 5.3 (0–14.8)  10.3 ± 20.8 | 11.9 (0–45.5)  31.2 ± 48.8 |
| 48­–72 h | N=377 | 115. 2 (60.9–199.0)  152.2 ± 157.1 | 0 (0­–0)  7.0 ± 49.1 | 0 (0–0)  9.2 ± 53.5 | 0 (0–0)  4.5 ± 20.6 | 0 (0­–0)  21.4 ± 54.1 | 0 (0–3.0)  13.6 ± 31.3 | 0 (0–0)  10.5 ± 31.7 | 15.4 (0–46.2)  27.2 ± 37.1 | 5.6 (0–14.8)  10.3 ± 21.3 | 5.6 (0–14.8)  10.3 ± 21.3 | 11.9 (0.6–41.7)  29.6 ± 46.2 |
| 72–96 h | N=318 | 94.3 (46.4–163.5)  125.9 ± 125.6 | 0 (0–0)  3.9 ± 22.9 | 0 (0–0)  7.2 ± 42.6 | 0 (0–0)  2.3 ± 12.1 | 0 (0­–0)  14.8 ± 44.1 | 0 (0–0)  11.1 ± 25.9 | 0 (0–0)  11.6 ± 33.9 | 15.4 (0–38.5)  23.9 ± 29.2 | 4.2 (0–13.8)  8.4 ± 12.1 | 0 (0-0.1)  3.6 ± 8.5 | 10.9 (0–35.4)  24.7 ± 36.5 |
| 96–120 h | N=265 | 87.1 (40.8–152.3)  118.1 ± 124.8 | 0 (0–0)  6.3 ± 34.9 | 0 (0–0)  7.8 ± 53.0 | 0 (0–0)  3.2 ± 15.0 | 0 (0­–0)  13.3 ± 46.5 | 0 (0–0)  9.9 ± 24.7 | 0 (0–0)  14.0 ± 43.5 | 12.3 (0–30.8)  21.3 ± 28.3 | 3.7 (0–13.4)  8.7 ± 12.4 | 0 (0–0.1)  4.3 ± 20.2 | 7.7 (0–26.9)  21.5 ± 37.1 |
| 120–144 h | N=200 | 76.0 (39.2–141.7)  110.0 ± 118.8 | 0 (0–0)  1.9 ± 10.7 | 0 (0–0)  8.8 ± 53.9 | 0 (0–0)  3.0 ± 15.3 | 0 (0­–0)  7.6 ± 32.3 | 0 (0–0)  6.1 ± 19.7 | 0 (0–0.6)  16.8 ± 46.4 | 9.8 (0–30.8)  19.4 ± 26.1 | 3.8 (0–14.8)  9.2 ± 13.0 | 0 (0–1.3)  3.1 ± 6.9 | 11.7 (0–33.6)  22.3 ± 31.5 |
| 144–168 h | N=170 | 81.3 (39.0–151.2)  114.0 ± 129.5 | 0 (0–0)  2.0 ± 14.7 | 0 (0–0)  10.0 ± 71.3 | 0 (0–0)  3.8 ± 16.3 | 0 (0­–0)  13.1 ± 43.4 | 0 (0–0)  4.7 ± 13.5 | 0 (0–0)  17.4 ± 46.1 | 9.2 (0–30.8)  21.1 ± 31.9 | 2.9 (0–14.8)  9.5 ± 14.1 | 0 (0–0)  3.1 ± 8.3 | 8.5 (0–25.2)  19.5 ± 31.5 |

The amount of each fluid was presented as a median (interquartile range) and mean ± standard deviation.

Abbreviations: ICU, intensive care unit; KVO, keep venous open.

1. Patients without hypoxemia (P/F > 300)

| ICU stay | No. of patients | Total fluid, mEq | Resuscitation | Blood product, mEq | Colloid, mEq | Maintenance | | Nutrition, mEq | Fluid creep | | | |
| --- | --- | --- | --- | --- | --- | --- | --- | --- | --- | --- | --- | --- |
|  |  |  | Isotonic crystalloid, mEq |  |  | Isotonic crystalloid, mEq | Hypotonic crystalloid, mEq |  | Antibiotics, mEq | Sedation / analgesia, mEq | Vasoactive drug, mEq | Miscellaneous use, mEq |
| –24 h | N=167 | 228.5 (146.6–401.5)  309.9 ± 264.6 | 0 (0–54.5)  67.8 ± 165.0 | 0 (0–0)  24.1 ± 61.0 | 0 (0–0)  17.3 ± 60.0 | 90.8 (0–134.3)  92.1 ± 97.6 | 0 (0–26.8)  19.3 ± 36.7 | 0 (0–0)  2.1 ± 9.8 | 30.8 (0–46.2)  26.8 ± 25.9 | 5.7 (0–13.4)  9.4 ± 11.5 | 0 (0–1.4)  3.4 ± 11.1 | 10.6 (0–47.7)  27.9 ± 38.4 |
| 24–48 h | N=111 | 157.4 (90.1–230.1)  193.7 ± 176.1 | 0 (0­–0)  15.4 ± 74.1 | 0 (0–0)  8.8 ± 35.2 | 0 (0–0)  6.8 ± 21.7 | 0 (0–86.0)  49.5 ± 86.2 | 0 (0–13.8)  13.9 ± 28.7 | 0 (0–0)  6.8 ± 22.1 | 30.8 (0–46.2)  28.5 ± 25.1 | 4.1 (0–14.1)  12.2 ± 37.4 | 4.1 (0–14.1)  12.1 ± 37.4 | 7.7 (0–52.8)  34.2 ± 59.5 |
| 48­–72 h | N=75 | 125.5 (65.8–199.0)  154.0 ± 134.0 | 0 (0­–0)  4.8 ± 41.3 | 0 (0–0)  2.4 ± 10.7 | 0 (0–0)  4.7 ± 24.3 | 0 (0–10.5)  27.7 ± 57.5 | 0 (0–6.2)  13.1 ± 26.3 | 0 (0–0)  13.0 ± 35.2 | 15.4 (0–46.2)  26.0 ± 29.2 | 1.4 (0–11.9)  11.3 ± 39.9 | 1.4 (0–11.9)  11.3 ± 39.9 | 10.8 (0–52.4)  33.3 ± 55.3 |
| 72–96 h | N=59 | 127.3 (60.1–190.2)  147.1 ± 121.2 | 0 (0­–0)  5.8 ± 31.2 | 0 (0–0)  4.2 ± 19.0 | 0 (0–0)  2.2 ± 11.9 | 0 (0–24.3)  28.7 ± 60.3 | 0 (0–0)  12.2 ± 24.4 | 0 (0–4.5)  18.0 ± 43.5 | 15.4 (0–32.9)  22.5 ± 24.9 | 0 (0­–7.4)  5.8 ± 10.0 | 0 (0–0)  2.0 ± 6.7 | 18.5 (0–44.6)  32.2 ± 52.2 |
| 96–120 h | N=45 | 113.2 (61.0–161.1)  139.7 ± 119.0 | 0 (0­–0)  3.8 ± 18.6 | 0 (0–0)  2.8 ± 13.2 | 0 (0–0)  2.2 ± 10.8 | 0 (0­–0)  28.4 ± 64.4 | 0 (0–0)  8.8 ± 17.7 | 0 (0–16.8)  20.9 ± 63.0 | 12.9 (0–30.8)  22.9 ± 38.9 | 0 (0–7.4)  6.3 ± 11.3 | 0 (0–0)  2.7 ± 9.5 | 7.7 (0–37.0)  32.7 ± 58.8 |
| 120–144 h | N=34 | 86.3 (43.5–161.2)  118.0 ± 113.3 | 0 (0­–0)  1.3 ± 7.8 | 0 (0–0)  0 ± 0 | 0 (0–0)  1.8± 10.3 | 0 (0­–0)  23.5 ± 63.6 | 0 (0–0)  5.4 ± 12.8 | 0 (0–16.8)  27.5 ± 75.5 | 3.1 (0–23.1)  17.9 ± 33.0 | 0 (0­–8.2)  7.2 ± 12.1 | 0 (0–0)  1.1 ± 3.5 | 7.7 (0–37.0)  23.0 ± 31.9 |
| 144–168 h | N=25 | 108.8 (17.8–156.8)  111.5 ± 102.4 | 0 (0­–0)  2.2 ± 10.9 | 0 (0–0)  0 ± 0 | 0 (0–0)  6.0 ± 20.9 | 0 (0–43.5)  37.0 ± 75.4 | 0 (0–0)  7.1 ± 13.5 | 0 (0–0)  18.4 ± 56.0 | 0 (0–10.1)  9.9 ± 18.1 | 0 (0­–7.4)  4.8 ± 8.6 | 0 (0–0)  1.1 ± 3.9 | 3.1 (0–15.4)  14.1 ± 27.5 |

The amount of each fluid was presented as a median (interquartile range) and mean ± standard deviation.

Abbreviations: ICU, intensive care unit; KVO, keep venous open.

1. Patients with mild hypoxemia (150 < P/F ≤ 300)

| ICU stay | No. of patients | Total fluid, mEq | Resuscitation | Blood product, mEq | Colloid, mEq | Maintenance | | Nutrition, mEq | Fluid creep | | | |
| --- | --- | --- | --- | --- | --- | --- | --- | --- | --- | --- | --- | --- |
|  |  |  | Isotonic crystalloid, mEq |  |  | Isotonic crystalloid, mEq | Hypotonic crystalloid, mEq |  | Antibiotics, mEq | Sedation / analgesia, mEq | Vasoactive drug, mEq | Miscellaneous use, mEq |
| –24 h | N=297 | 226.3 (131.3–356.4)  287.3 ± 275.4 | 0 (0–55.0)  61.4 ± 130.2 | 0 (0–30.8)  38.4 ± 129.7 | 0 (0–0)  12.7 ± 29.3 | 54.5 (0–124.8)  78.5 ± 94.2 | 0 (0–0)  9.5 ± 24.2 | 0 (0–0)  1.9 ± 11.1 | 15.4 (0–37.2)  28.1 ± 83.9 | 5.5 (0­–13.4)  7.8 ± 8.9 | 0 (0­–11.1)  7.8 ± 15.1 | 10.6 (0.2–43.5)  28.9 ± 42.6 |
| 24–48 h | N=247 | 132.2 (71.7–209.4)  172.5 ± 172.9 | 0 (0­–0)  15.2 ± 70.1 | 0 (0-0)  11.5 ± 52.2 | 0 (0–0)  3.6 ± 16.9 | 0 (0–57.8)  41.2 ± 74.6 | 0 (0–0)  10.4 ± 27.5 | 0 (0–0)  6.3 ± 32.8 | 17.2 (3.1–36.1)  24.7 ± 25.8 | 4.8 (0–14.8)  8.7 ± 11.0 | 4.8 (0–14.8)  8.7 ± 11.0 | 12.2 (0–43.1)  31.5 ± 49.6 |
| 48­–72 h | N=199 | 109.7 (53.9–190.7)  151.8 ± 179.3 | 0 (0­–0)  8.2 ± 57.8 | 0 (0-0)  13.1 ± 68.1 | 0 (0–0)  4.9 ± 22.7 | 0 (0­–0)  18.6 ± 44.2 | 0 (0–0)  12.5 ± 32.6 | 0 (0–0)  10.3 ± 32.9 | 15.4 (0–37.8)  25.9 ± 39.4 | 4.1 (0–13.5)  8.9 ± 12.6 | 4.1 (0–13.3)  8.8 ± 12.6 | 10.8 (0–47.5)  29.6 ± 48.6 |
| 72–96 h | N=168 | 80.7 (41.1–151.9)  120.0 ± 138.3 | 0 (0­–0)  3.6 ± 21.2 | 0 (0-0)  9.4 ± 54.5 | 0 (0–0)  3.5 ± 15.0 | 0 (0­–0)  9.3 ± 33.8 | 0 (0–0)  10.4 ± 27.6 | 0 (0–0)  11.0 ± 34.0 | 15.3 (0–30.8)  22.3 ± 27.3 | 4.8 (0–13.3)  7.8 ± 9.8 | 0 (0–2.5)  3.7 ± 7.5 | 10.1 (0–33.6)  22.6 ± 32.3 |
| 96–120 h | N=141 | 76.5 (31.4–137.0)  114.6 ± 142.0 | 0 (0­–0)  8.5 ± 44.4 | 0 (0-0)  10.8 ± 70.7 | 0 (0–0)  4.5 ± 18.8 | 0 (0­–0)  11.0 ± 44.7 | 0 (0–0)  7.1 ± 20.4 | 0 (0–0)  14.1 ± 43.4 | 9.2 (0–30.8)  19.2 ± 24.4 | 2.7 (0–13.4)  7.9 ± 10.9 | 0 (0–0.6)  5.1 ± 26.3 | 5.5 (0–20.0)  18.3 ± 32.9 |
| 120–144 h | N=108 | 66.7 (35.8–135.6)  108.9 ± 136.8 | 0 (0­–0)  2.5 ± 12.8 | 0 (0-0)  15.1 ± 72.4 | 0 (0–0)  3.8 ± 15.8 | 0 (0­–0)  5.4 ± 21.6 | 0 (0–0)  4.1 ± 13.5 | 0 (0–0)  16.0 ± 42.9 | 9.2 (0–25.8)  17.2 ± 21.4 | 3.3 (0–14.2)  8.3 ± 11.6 | 0 (0–2.1)  3.3 ± 6.7 | 9.5 (0–25.2)  20.4 ± 34.2 |
| 144–168 h | N=98 | 76.1 (31.6–140.0)  111.4 ± 148.8 | 0 (0­–0)  2.9 ± 18.5 | 0 (0-0)  16.3 ± 93.4 | 0 (0–0)  3.9 ± 17.2 | 0 (0­–0)  11.4 ± 39.0 | 0 (0–0)  3.3 ± 11.4 | 0 (0–8.2)  16.6 ± 43.4 | 9.2 (0–24.6)  17.6 ± 22.4 | 2.6 (0–12.1)  8.6 ± 12.8 | 0 (0–1.1)  3.3 ± 7.1 | 7.4 (0–18.5)  18.6 ± 33.3 |

The amount of each fluid was presented as a median (interquartile range) and mean ± standard deviation.

Abbreviations: ICU, intensive care unit; KVO, keep venous open.

1. Patients with severe hypoxemia (P/F ≤ 150)

| ICU stay | No. of patients | Total fluid, mEq | Resuscitation | Blood product, mEq | Colloid, mEq | Maintenance | | Nutrition, mEq | Fluid creep | | | |
| --- | --- | --- | --- | --- | --- | --- | --- | --- | --- | --- | --- | --- |
|  |  |  | Isotonic crystalloid, mEq |  |  | Isotonic crystalloid, mEq | Hypotonic crystalloid, mEq |  | Antibiotics, mEq | Sedation / analgesia, mEq | Vasoactive drug, mEq | Miscellaneous use, mEq |
| –24 h | N=124 | 239.0 (102.3–412.6)  308.5 ± 285.3 | 0 (0–54.5)  42.1 ± 111.4 | 0 (0–0)  51.5 ± 192.3 | 0 (0–0)  20.8 ± 52.5 | 12.0 (0–136.7)  77.9 ± 98.2 | 0 (0–0)  13.5 ± 30.5 | 0 (0–0)  3.6 ± 14.5 | 15.4 (0–46.2)  31.5 ± 37.5 | 6.9 (0–15.5)  10.2 ± 11.4 | 0 (0–16.4)  10.1 ± 15.7 | 15.3 (3.1–45.1)  29.2 ± 34.0 |
| 24–48 h | N=111 | 151.7 (79.8–244.3)  192.0 ± 170.5 | 0 (0­–0)  7.3 ± 31.7 | 0 (0–0)  18.9 ± 80.7 | 0 (0–0)  5.2 ± 17.9 | 0 (0–62.3)  47.3 ± 92.0 | 0 (0–13.4)  15.8 ± 33.1 | 0 (0–0)  7.2 ± 23.1 | 15.4 (3.1–46.2)  29.8 ± 46.1 | 8.3 (0–16.7)  11.9 ± 13.0 | 8.3 (0–16.7)  11.8 ± 13.0 | 15.4 (2.3–44.9)  27.7 ± 32.5 |
| 48­–72 h | N=103 | 121.1 (64.5–204.9)  151.6 ± 124.9 | 0 (0­–0)  6.4 ± 34.1 | 0 (0–0)  6.6 ± 37.4 | 0 (0–0)  3.4 ± 11.7 | 0 (0–0)  22.2 ± 67.3 | 0 (0–28.1)  16.2 ± 32.3 | 0 (0–0)  9.1 ± 26.2 | 15.4 (0–46.2)  30.7 ± 37.7 | 9.2 (0–17.0)  12.5 ± 14.2 | 9.2 (0–17.0)  12.5 ± 14.2 | 13.5 (2.2–38.8)  26.9 ± 32.4 |
| 72–96 h | N=91 | 95.8 (50.5–165.1)  123.1 ± 101.1 | 0 (0­–0)  3.4 ± 19.8 | 0 (0–0)  5.1 ± 24.9 | 0 (0–0)  0.0 ± 0.5 | 0 (0–0)  16.0 ± 47.0 | 0 (0–8.4)  11.5 ± 23.7 | 0 (0–0)  8.4 ± 25.3 | 15.4 (0–46.2)  30.7 ± 37.7 | 7.4 (0–14.8)  11.3 ± 16.0 | 0 (0–2.1)  4.7 ± 10.8 | 12.1 (1.4–30.6)  23.7 ± 31.1 |
| 96–120 h | N=79 | 90.9 (45.1–156.4)  112.2 ± 90.4 | 0 (0­–0)  4.0 ± 18.8 | 0 (0–0)  5.3 ± 20.0 | 0 (0–0)  1.4 ± 7.4 | 0 (0–0)  8.8 ± 35.0 | 0 (0–13.2)  15.4 ± 33.3 | 0 (0–0)  9.9 ± 27.1 | 15.4 (0–46.2)  27.7 ± 34.7 | 6.1 (0–14.8)  11.2 ± 15.0 | 0 (0–2.6)  3.9 ± 9.2 | 12.3 (1.8–27.7)  20.7 ± 26.0 |
| 120–144 h | N=58 | 87.6 (44.5–159.1)  107.4 ± 81.6 | 0 (0­–0)  1.2 ± 7.6 | 0 (0–0)  2.0 ± 10.8 | 0 (0–0)  2.2 ± 17.1 | 0 (0–0)  2.4 ± 15.7 | 0 (0–0)  10.2 ± 29.8 | 0 (0–0)  12.1 ± 25.7 | 15.4 (0–46.2)  24.1 ± 27.6 | 7.4 (0–17.3)  11.9 ± 15.4 | 0 (0–4.8)  3.9 ± 8.3 | 20.4 (2.3–37.0)  25.5 ± 25.5 |
| 144–168 h | N=47 | 96.1 (49.6–191.9)  120.9 ± 96.7 | 0 (0­–0)  0 ± 0 | 0 (0–0)  2.3 ± 8.7 | 0 (0–0)  2.6 ± 11.0 | 0 (0–0)  3.8 ± 18.3 | 0 (0–0)  6.4 ± 17.1 | 0 (0–0)  18.6 ± 47.1 | 15.4 (0–34.7)  24.3 ± 29.2 | 7.8 (0–20.3)  14.0 ± 17.6 | 0 (0–0)  3.8 ± 11.7 | 16.8 (0–33.6)  24.2 ± 29.4 |

The amount of each fluid was presented as a median (interquartile range) and mean ± standard deviation.

Abbreviations: ICU, intensive care unit; KVO, keep venous open.

**Table S4** Estimates of the effects of covariates on the fluid volume, sodium, and chloride in the multiple linear regression models

1. Fluid volume

|  | Total intravenous fluid, mL (95% CI) | *P* value | Fluid creep, mL (95% CI) | *P* value |
| --- | --- | --- | --- | --- |
| Male | -256 (-638.7 to -126.7) | 0.189 | -19.4 (-141.6 to 102.8) | 0.755 |
| Age, per 5 y | -59.0 (-133.3 to 15.3) | 0.120 | -36.3 (-60.1 to -12.6) | 0.003 |
| Body mass index, kg/m^2^ | -25.2 (-68.2 to 17.9) | 0.251 | 7.5 (-6.2 to 21.3) | 0.283 |
| Emergency admission | -1114.2 (-1589.6 to -638.9) | <0.001 | -123.0 (-274.8 to 28.8) | 0.112 |
| Cardiovascular disease | -127.6 (-565.0 to 309.8) | 0.567 | 50.8 (-88.9 to 190.5) | 0.476 |
| APACHE II score, per 5 points | 0.3 (-149.8 to 150.5) | 0.996 | -32.5 (-80.5 to 15.5) | 0.184 |
| SOFA score, per 1 point | 174.3 (99.9 to 248.8) | <0.001 | 46.8 (23.1 to 70.6) | <0.001 |
| Charlson comorbidity index, per 1 point | -94.3 (-183.2 to -5.3) | 0.038 | 7.0 (-21.5 to 35.4) | 0.631 |
| Sepsis | -64.4 (-746.3 to 617.4) | 0.853 | 73.8 (-143.9 to 291.6) | 0.506 |
| Septic shock | 1756.9 (1123.9 to 2390.0) | <0.001 | 249.8 (47.6 to 452.0) | 0.016 |
| Hypoxemic respiratory failure | -467.2 (-913.8 to -20.5) | 0.040 | -50.1 (-192.8 to 92.5) | 0.490 |

Adjusted by the following factors: sex, age, body mass index, emergency admission, cardiovascular disease, APACHE II score, SOFA score, Charlson comorbidity index, sepsis, septic shock, hypoxemic respiratory failure.

Abbreviations: APACHE, acute physiology and chronic health evaluation; CI, confidence interval; SOFA, sequential organ failure assessment.

**Table S4** Estimates of the effects of covariates on the fluid volume, sodium, and chloride in the multiple linear regression models

1. Sodium burden

|  | Total sodium burden, mEq (95% CI) | *P* value | Sodium burden due to fluid creep, mEq (95% CI) | *P* value |
| --- | --- | --- | --- | --- |
| Male | 49.6 (-1.1 to 100.3) | 0.055 | 5.7 (-7.8 to 19.3) | 0.405 |
| Age, per 5 y | -0.3 (-10.2 to 9.5) | 0.945 | -0.4 (-3.1 to 2.2) | 0.738 |
| Body mass index, kg/m^2^ | -2.9 (-8.6 to 2.8) | 0.321 | 0.8 (-0.7 to 2.3) | 0.290 |
| Emergency admission | -100.9 (-163.9 to -37.9) | 0.002 | -6.2 (-23.1 to 10.6) | 0.4666 |
| Cardiovascular disease | -29.3 (-87.2 to 28.7) | 0.322 | -10.0 (-25.6 to 5.37) | 0.201 |
| APACHE II score, per 5 points | -2.46 (-22.4 to 17.4) | 0.808 | -5.6 (-10.9 to -0.3) | 0.038 |
| SOFA score, per 1 point | 26.3 (16.4 to 36.1) | <0.001 | 4.9 (2.2 to 7.5) | <0.001 |
| Charlson comorbidity index, per 1 point | -14.0 (-25.8 to -2.3) | 0.020 | -0.2 (-3.4 to 2.9) | 0.885 |
| Sepsis | 28.1 (-62.2 to 118.5) | 0.541 | -1.1 (-25.2 to 23.0) | 0.927 |
| Septic shock | 227.0 (143.1 to 310.9) | <0.001 | 14.0 (-8.4 to 36.4) | 0.220 |
| Hypoxemic respiratory failure | -74.0 (-133.2 to -14.8) | 0.014 | -0.9 (-16.7 to 14.9) | 0.913 |

Adjusted by the following factors: sex, age, body mass index, emergency admission, cardiovascular disease, APACHE II score, SOFA score, Charlson comorbidity index, sepsis, septic shock, hypoxemic respiratory failure.

Abbreviations: APACHE, acute physiology and chronic health evaluation; CI, confidence interval; SOFA, sequential organ failure assessment.

**Table S4** Estimates of the effects of covariates on the fluid volume, sodium, and chloride in the multiple linear regression models

1. Chloride burden

|  | Total chloride burden, mEq (95% CI) | *P* value | Chloride burden due to fluid creep, mEq (95% CI) | *P* value |
| --- | --- | --- | --- | --- |
| Male | 44.6 (0.3 to 89.0) | 0.048 | 13.7 (-0.1 to 27.6) | 0.052 |
| Age, per 5 y | -0.3 (-9.0 to 8.3) | 0.938 | -0.9 (-3.6 to 1.8) | 0.510 |
| Body mass index, kg/m^2^ | -2.5 (-7.5 to 2.5) | 0.322 | 1.1 (-0.5 to 2.63) | 0.174 |
| Emergency admission | -91.6 (-146.7 to -36.5) | 0.001 | -10.2 (-27.4 to 6.9) | 0.242 |
| Cardiovascular disease | -24.0 (-74.7 to 26.6) | 0.352 | -7.9 (-23.8 to 7.9) | 0.324 |
| APACHE II score, per 5 points | -3.4 (-20.8 to 14.0) | 0.702 | -5.7 (-11.1 to -0.3) | 0.039 |
| SOFA score, per 1 point | 22.9 (14.2 to 31.5) | <0.001 | 5.9 (3.2 to 8.5) | <0.001 |
| Charlson comorbidity index, per 1 point | -12.4 (-22.7 to -2.1) | 0.018 | -1.5 (-4.7 to 1.7) | 0.354 |
| Sepsis | 21.3 (-57.7 to 100.2) | <0.001 | 9.9 (34.5 to -14.7) | 0.430 |
| Septic shock | 194.8 (121.5 to 268.2) | <0.001 | 25.3 (2.5 to 48.2) | 0.030 |
| Hypoxemic respiratory failure | -61.4 (-113.1 to -9.7) | 0.020 | -3.6 (-19.7 to 12.6) | 0.663 |

Adjusted by the following factors: sex, age, body mass index, emergency admission, cardiovascular disease, APACHE II score, SOFA score, Charlson comorbidity index, sepsis, septic shock, hypoxemic respiratory failure.

Abbreviations: APACHE, acute physiology and chronic health evaluation; CI, confidence interval; SOFA, sequential organ failure assessment.
